# Molecular Tailoring of Pyridine Core-Based Hole Selective Layer for Lead Free Double Perovskite Solar Cells Fabrication

**DOI:** 10.1021/acsaem.3c01027

**Published:** 2023-07-17

**Authors:** Peng Huang, Manju Sheokand, David Payno Zarceño, Samrana Kazim, Luis Lezama, Mohammad Khaja Nazeeruddin, Rajneesh Misra, Shahzada Ahmad

**Affiliations:** † BCMaterials, Basque Center for Materials, Applications and Nanostructures, Martina Casiano, UPV/EHU Science Park, 48940 Leioa, Spain; ‡ Department of Chemistry, Indian Institute of Technology, 453552 Indore, India; § Departamento de Química Inorgánica, Facultad de Ciencia y Tecnología, Universidad del País Vasco, UPV/EHU, Sarriena s/n, 48940 Leioa, Spain; ∥ Group for Molecular Engineering of Functional Materials, Institute of Chemical Sciences and Engineering, École Polytechnique Fedérale de Lausanne, 1951 Sion, Switzerland; ⊥ IKERBASQUE, Basque Foundation for Science, 48009 Bilbao, Spain; # Research Institute of Frontier Science, Southwest Jiaotong University, 610031 Chengdu, China

**Keywords:** Cs_2_AgBiBr_6_, small organic molecule, perovskite solar cells, electron paramagnetic resonance, photovoltaic simulation

## Abstract

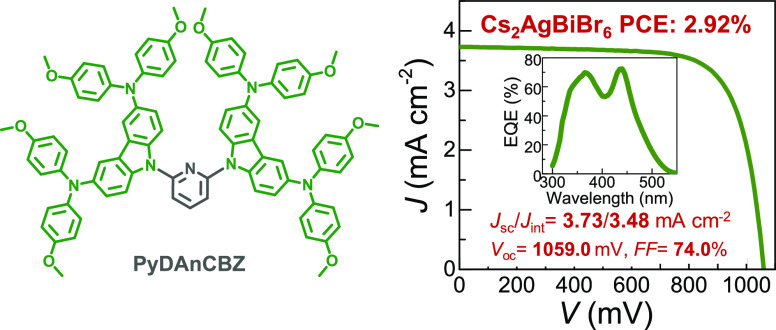

To solve the toxicity issues related to lead-based halide perovskite solar cells, the lead-free double halide perovskite Cs_2_AgBiBr_6_ is proposed. However, reduced rate of charge transfer in double perovskites affects optoelectronic performance. We designed a series of pyridine-based small molecules with four different arms attached to the pyridine core as hole-selective materials by using interface engineering. We quantified how arm modulation affects the structure–property–device performance relationship. Electrical, structural, and spectroscopic investigations show that the *N*^3^,*N*^3^,*N*^6^,*N*^6^-tetrakis(4-methoxyphenyl)-9*H*-carbazole-3,6-diamine arm’s robust association with the pyridine core results in an efficient hole extraction for **PyDAnCBZ** due to higher spin density close to the pyridine core. The solar cells fabricated using Cs_2_AgBiBr_6_ as a light harvester and **PyDAnCBZ** as the hole selective layer measured an unprecedented 2.9% power conversion efficiency. Our computed road map suggests achieving ∼5% efficiency through fine-tuning of Cs_2_AgBiBr_6_. Our findings reveal the principles for designing small molecules for electro-optical applications as well as a synergistic route to develop inorganic lead-free perovskite materials for solar applications.

## 1 Introduction

Lead halide perovskite has shown remarkable optoelectrical and photovoltaics properties, and solution-processibility offers cost-effective deposition routes, arguably increasing its acceptability as a potential candidate for next-generation photovoltaics (PV).^1−4^ However, its poor stability and the toxicity of lead restrict its immediate commercial endeavor.^5−8^ To close this gap, significant attempts are being made to exploit interface materials to improve stability,^9,10^ and the development of an eco-friendly perovskite for PV.^11,12^

Double metal perovskite (Cs_2_AgBiBr_6_) is an attractive alternative for thin-film PV, attributed to its enhanced thermal stability, less toxicity, and favorable photoelectrical traits including long carrier lifespan and moderate charge carrier mobility.^13−15^ Efforts are being made to enhance the performance of Cs_2_AgBiBr_6_-based solar cells. This includes innovative deposition processes,^14,16,17^ dye sensitization,^13,18,19^ additive engineering,^20−22^ binary solvent,^14,23^ and interface engineering^24−27^ to assist high-quality film formation, which in turn facilitate carrier transport, and thus power conversion efficiency (PCE). Despite these advancements, the PCEs are far below compared to the Pb-based perovskites with comparable bandgaps. This entails new approaches in the investigation to boost PV performance.

The competitive performance of Cs_2_AgBiBr_6_ solar cells has been achieved by the typical device architect, i.e., FTO/ESL/Cs_2_AgBiBr_6_/HSL/Au (electron and hole selective layers are abbreviated as ESL and HSL). Among them, 2,2′,7,7′-tetrakis-(*N*,*N*-di-*p*-methoxyphenyl-amine)-9,9′-spirobifluorene (Spiro-OMeTAD) provides facile implementation and is the widely investigated HSL.^28^ However, the compulsory hydrophilic doping of Spiro diminishes the stability merit of Cs_2_AgBiBr_6_. Additionally, the poor transport capacity of Spiro-OMeTAD will aid carrier recombination. Arguably, the development of functionalized HSLs is paramount for Cs_2_AgBiBr_6_ for performance and stability enhancement.

Small molecules with unique features offer prospective for application in lead-free Cs_2_AgBiBr_6_: low batch-to-batch variance, diversity via molecular design methodologies, and cost-effectiveness.^29^ The pyridine can be served as an electron-deficient and passivation function unit to create new HSLs.^30^ Pyridine-based HSLs and associated synthesis methodologies have been reported.^31,32^ We put forward the linking topology of the pyridine core and common arms, as well as the *N*-position effects on pyridine-based HSLs.^33,34^

The arm modulation based on pyridine-based HSLs has not been probed yet. Notably, the incorporation of innovative small organic molecule as HSLs in Cs_2_AgBiBr_6_ perovskite based solar cells remains unexplored. Here, we developed four pyridine-based HSLs using certain arms in the 2,6-position linking of pyridine: *N*,*N*-di-4-anisylamino (DAn), (*N*,*N*-bis(4-methoxyphenyl)aniline) (PDAn), (*N*^3^,*N*^3^,*N*^6^,*N*^6^-tetrakis(4-methoxyphenyl)-9*H*-carbazole-3,6-diamine) (DAnCBZ), and 4,4′-(10*H*-phenothiazine-3,7-diyl)bis(*N*,*N*-bis(4-methoxyphenyl)-aniline) (PTPDAn), named as **PyDAn**, **PyPDAn**, **PyDAnCBZ**, and **PyPTPDAn**, respectively. We employed an array of techniques to quantify the chemical and optoelectronic properties. Moreover, we fabricated the Cs_2_AgBiBr_6_-based solar cells employing pyridine-based HSLs, and the **PyDAnCBZ**-based devices showed superior performance than other HSLs. Furthermore, we computed the photovoltaic simulation to unravel the road map for the lead-free device performance enhancement method.

## 2 Results and Discussion

### 2.1 Synthesis

**Scheme 1:**
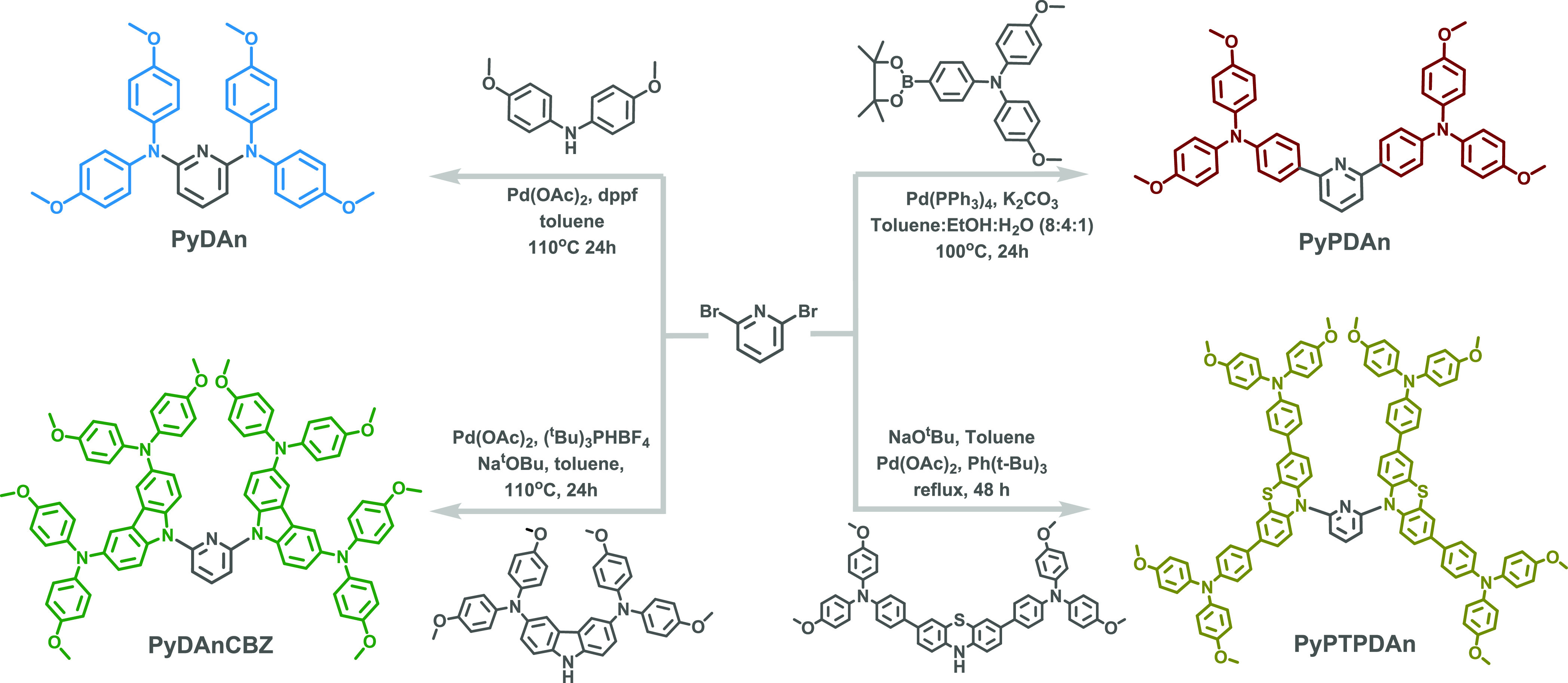
Synthetic Routes for **PyDAn**, **PyPDAn**, **PyDAnCBZ**, and **PyPTPDAn**

Herein, we critically analyze arm modulation employing small molecules with a pyridine core and its 2,6-position linking topology. The DAn, PDAn, and DAnCBZ moieties have frequently been utilized as arms in the design of organic hole-selective materials.^35^ Moreover, PTPDAn, which consists of phenothiazine interacting with two PDAn arms, is the largest arm molecule. The synthetic routes of these materials are illustrated in Scheme 1. Four precursors were made using prior procedures that are detailed in the Supporting Information. The **PyDAn**, **PyDAnCBZ**, and **PyPTPDAn** were synthesized from the palladium acetate-catalyzed Buchwald-Hartwig cross-coupling reaction with yields of 70, 46, and 52%, respectively, while **PyPDAn** was synthesized by the Suzuki cross-coupling reaction using palladium-tetrakis(triphenylphosphine) as a catalyst and resulted in the final molecule with a yield of 60%. The molecular structures were established by ^1^H NMR, ^13^C NMR spectroscopy, and HRMS (Figure S1 in the Supporting Information). All the materials are soluble in common organic solvents.

### 2.2 Photophysical and Electrochemical Properties

**Figure 1:**
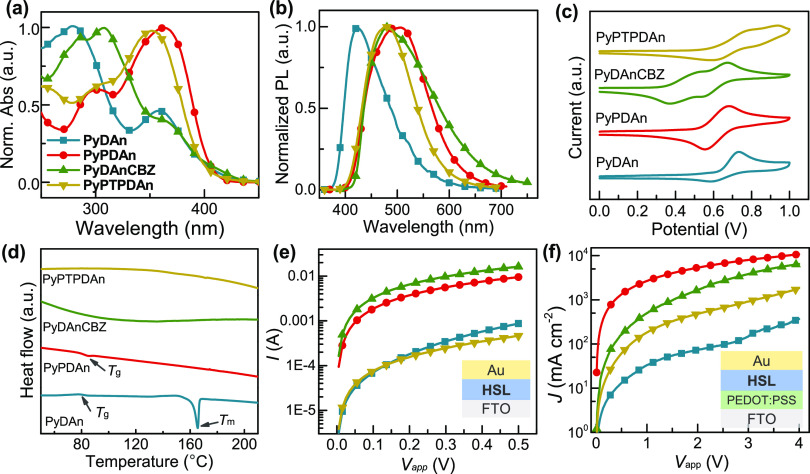
(a) Normalized UV–vis absorption spectra, (b) photoluminescent spectra, (c) cyclic voltammograms, and (d) differential scanning calorimetry curves of **PyDAn**, **PyPDAn**, **PyDAnCBZ**, and **PyPTPDAn**. (e) *I*–*V* curves for conductivity measurement, and (f) *J*–*V* curves of mobility measurement for the hole-only device with these HSLs.

The normalized optical absorption spectra of pyridine-based small molecules are presented (Figure 1a), and the data are compiled in Table 1. The spectra revealed two principal absorption bands: 250–310 and 340–420 nm. This is in line with previous results on other pyridine-based HSLs.^33,36^ The electronic transition causes an absorbed peak in the lower wavelength. The action of intramolecular charge transfer (ICT) between the armed group and electron-deficient pyridine core produced a substantial peak in the 340–420 nm region. According to the formula *E*_g_^opt^ = 1240/λ_onset_, the optical bandgap (*E*_g_^opt^) values were calculated to be 3.04, 3.06, 2.85, and 3.11 eV, respectively.

**Table 1:** Photophysical and Electrochemical Data of the **PyDAn**, **PyPDAn**, **PyDAnCBZ**, and **PyPTPDAn**

HSL	λ_onset_ (nm)	λ_abs.max_ (nm)	λ_PL max_ (nm)	Δλ_stokes_ (nm)	*E*_g_^opt^/*E*_g_^DFT^ (eV)	*E*^ox^_onset_ (eV)	*E*_HOMO_ (eV)	*E*_LUMO_ (eV)	*T*_d_ (°C)	*T*_g_ (°C)	cond. (μS cm^–1^)	mob. (10^–4^ cm^2^ V^–1^ s^–1^)
**PyDAn**	409	278	423	145	3.04/4.30	0.62	–5.02^a^/–5.72^b^	–2.60^a^/–0.30^b^	81.0	307.5	0.11	0.35
**PyPDAn**	405	363	506	143	3.06/4.19	0.54	–4.94^a^/–5.64^b^	–2.47^a^/–0.98^b^	82.0	372.5	1.23	1.85
**PyDAnCBZ**	436	308	480	172	2.85/3.30	0.36	–4.76^a^/–5.45^b^	–2.69^a^/–0.85^b^		242.0	2.11	3.50
**PyPTPDAn**	399	352	480	128	3.11/3.45	0.60	–5.00^a^/–5.70^b^	–2.35^a^/–1.46^b^		196.6	0.06	1.14

a *E*_HOMO_ was estimated from the redox potential in the CV curves, and *E*_LUMO_ = *E*_HOMO_ + *E*_g_^opt^.

b *E*_HOMO_ and *E*_LUMO_ were calculated by DFT results.

It can be deduced from Figure 1b that the peak maxima in photoluminescence spectra for **PyDAn**, **PyPDAn**, **PyDAnCBZ**, and **PyPTPDAn** appears at 423, 506, 480, and 480 nm, with Stokes shifts of 145, 143, 172, and 128 nm, respectively. For organic materials, larger Stokes shifts result from a shift in the material’s structure between the ground and excited states. It was observed that **PyDAnCBZ** has a larger Stokes shift than the others, which occurs from the modification of the geometrical configuration generated by fluorescence illumination and can enhance the capacity to fill pores, leading to higher charge transporting ability.^37,38^

The energy levels of HSLs were measured using cyclic voltammetry (Figure 1c and Figure S2). The **PyDAn** and **PyPDAn** showed only one reversible event, while the **PyDAnCBZ** and **PyPTPDAn** showed two reversible anodic events. Two DAn units connect to a carbazole in the **PyDAnCBZ**’s molecular configuration, and two PDAn units connect to a phenothiazine in **PyPTPDAn**’s, which may form stable dications owing to charge delocalization throughout the conjugation chain, whereas **PyDAn** and **PyPDAn** can only undergo single cation transformation. The **PyDAn**, **PyPDAn**, **PyDAnCBZ**, and **PyPTPDAn** have starting oxidation potentials of 0.62, 0.54, 0.36, and 0.60 eV, respectively. The **DAnCBZ** arm’s greater electron donation than other arms contributed to **PyDAnCBZ**’s lower oxidation potential. *E*_HOMO_ values of these compounds were −5.02, −4.94, −4.76, and −5.00 eV, calculated from *E*_HOMO_ = −4.4 + *E*^ox^_onset_. The corresponding *E*_LUMO_ was calculated by adding *E*_g_^opt^ to the *E*_HOMO_ and summarized in Table 1.

To unravel the energy level and geometrical structure of pyridine-based HSLs, we performed density functional theory (DFT) studies at the B3LYP/6-31G(d,p) (Figure S3). The *E*_LUMO_ and *E*_HOMO_ of **PyDAn** spread over the pyridine core and diphenylamine arms, and the *E*_HOMO_ of **PyPDAn** exhibits similarities in the electron densities with **PyDAn**, while the *E*_LUMO_ of **PyPDAn** is distributed on the central pyridine and benzene bridge. The *E*_HOMO_ of **PyDAnCBZ** was mainly distributed on DAnCBZ arms, and *E*_LUMO_ was mainly distributed on the pyridine core. The *E*_LUMO_ and *E*_HOMO_ were distributed on the pyridine core and PTPDAn arms in the **PyPTPDAn**.

To study the thermal properties of pyridine-based materials, differential scanning calorimetry (DSC) and thermogravimetric analysis (TGA) were utilized. TGA curves (Figure S4), the decomposition temperatures (*T*_d_) corresponding to 5% with loss temperature, were 307.5, 372.5, 242, and 196.6 °C for **PyDAn**, **PyPDAn**, **PyDAnCBZ**, and **PyPTPDAn**, respectively. It can be deduced that the *T*_d_ of pyridine-core based materials decreases as the arm size increases. The full and second heating run of DSC are presented (Figure S5 and Figure 1d). The **PyDAn** displays an evident glass-transition temperature (*T*_g_) at 81.0 °C and melting of the crystals (*T*_m_) at 165.4 °C, whereas **PyPDAn** displayed an apparent *T*_g_ at 82.0 °C. The **PyDAnCBZ** and **PyPTPDAn** did not have an obvious *T*_g_, showing their amorphous nature. The larger arms can enhance *T*_g_, and **PyDAnCBZ** (**PyPTPDAn**) has improved its thermal stability and is beneficial for success in the real operating conditions of solar cells.

The electrical measurements were carried out to deduce the impacts of the arm on the HSLs ability for charge transport. The conductivity of the HSLs were determined in a perpendicular configuration using the structure FTO/HSL/Au, and the *J*–*V* curves were measured in the dark. The σ value was determined from the slope of the *J*–*V* curves, using the equation σ = *JdV*^–1^, where *d* is the thickness of the HSL. The **PyDAn**, **PyDAn**, **PyDAnCBZ**, and **PyPTPDAn** had σ values of 0.11, 1.23, 2.11, and 0.06 μS cm^–1^, respectively (Figure 1e). Further, we evaluated the hole mobility of the synthesized HSLs using the space charge limited current (SCLC) method. A hole-only device structure of ITO/PEDOT:PSS/HTM/Ag was employed, and *J*–*V* curves were measured under dark and ambient conditions (Figure 1f). The hole mobility values of the HTMs were determined using the Mott-Gurney equation: *J* = 9εε_0_μ*V*_app_^2^/8 *L*^3^, where ε and ε_0_ represent the dielectric permittivity and dielectric constant, respectively. The obtained data are compiled in Table 1. The pristine HSLs exhibited hole mobilities of 0.35, 1.85, 3.50, and 1.14 (× 10^–4^ cm^2^ V^–1^ s^–1^). The highest charge transporting value of **PyDAnCBZ** as compared to others derives from the improved intramolecular charge transfer and uniform morphology, subsequently discussed.

**Figure 2:**
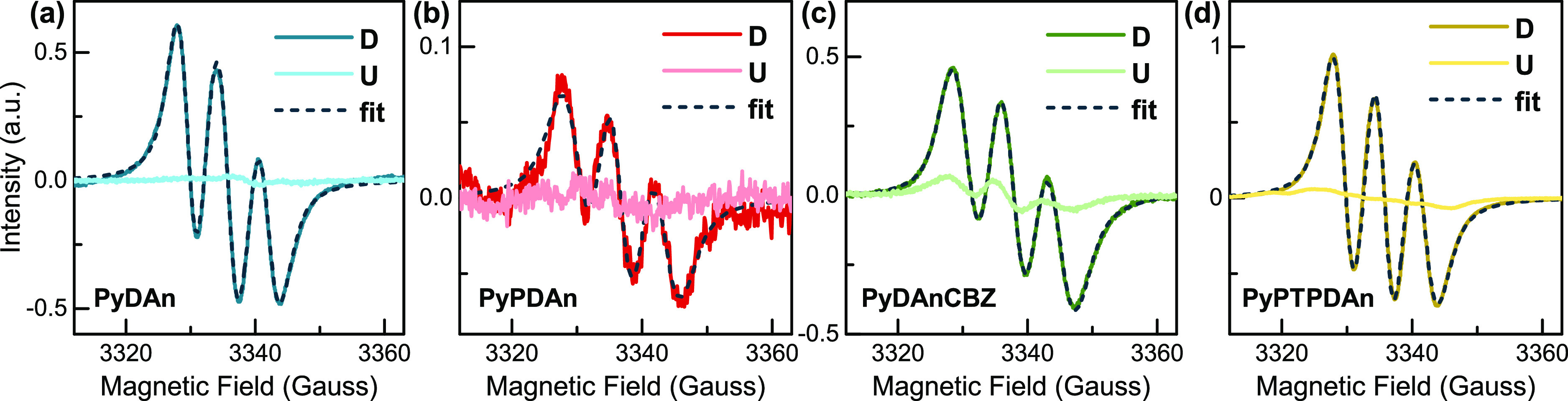
Room-temperature EPR spectra registered on 25 mM toluene solutions at the X-band. The dotted lines represent the best fits obtained using the spectral parameters listed in Table S1.

Electron paramagnetic resonance (EPR) spectroscopy is an advantageous technique to identify molecules with unpaired electrons and to evaluate the relative number of spins and their mobility. For this purpose, X-band EPR experiments on pristine and doped 25 mM toluene solutions of the four pyridine-based HSLs were performed at room temperature (Figure 2). The EPR signal of the undoped samples was difficult to detect due to the number of spins inside the resonant cavity being practically below the detection limit of the spectrometer (about 10^9^). In contrast, a dramatic enhancement of the signal was observed after the addition of lithium bis(trifluoromethanesulfonyl)imide (LiTFSI) and 4-*tert* butylpyridine (*t*-BP), confirming a remarkable number of holes in these materials after LiTFSI-doping (≈10^17^ spin/mol). The structure of the spectra, three equally spaced lines with intensity ratios close to 1:1:1, indicates that the unpaired electrons are preferentially located on ^14^N nuclei (*I* = 1), but the generation of other radicals with very short lifetime (<10^–6^ s) cannot be disregarded. The absence of proton hyperfine structure is attributed to the presence of many protons with small hyperfine couplings. The spectra could be fitted to obtain the *g* value, the coupling parameter *a^N^*, and the peak-to-peak linewidth (Table S1). The calculated g values (2.0044, 2.0040, 2.0037, and 2.0043 for **PyDAn**, **PyPDAn**, **PyDAnCBZ**, and **PyPTPDAn**, respectively) are in good agreement with those expected for N-centered cation radicals with the low orbital contribution to the ground state. The hyperfine coupling constant (*a^N^*) and the peak-to-peak linewidth are relatively larger for **PyDAnCBZ** (6.8 and 4.8 Gauss, respectively) than for the other compounds, suggesting a higher spin density near the pyridine core.

Before the fabrication of the device, the surface properties and film-formation capabilities of HSLs were investigated. The Cs_2_AgBiBr_6_ film was prepared using the hot-casting method, and as a result of the rapid crystallization, the as-prepared films (Figure S6) showed micrometer-sized agglomerates on the surface measured by scanning electron microscopy (SEM), which is in accordance with the results reported by Shao et al. and Bein et al.^14,39^ The images of perovskite with HSLs atop the perovskite were presented (Figure S7). The perovskite/**PyDAn** exhibited subpar surface morphologies, whereas others show uniform coverage. To evaluate the morphology properties of HSLs, the HSLs were deposited on flat substrates. It showed that the **PyDAnCBZ** film had a remarkably uniform, smooth, and nanoparticle-free surface. In contrast, **PyDAn** and **PyPTPDAn** films display nonuniform coverage, whereas **PyPDAn** displayed aggregation and undissolved dopants.

### 2.3 Device Performance

**Figure 3:**
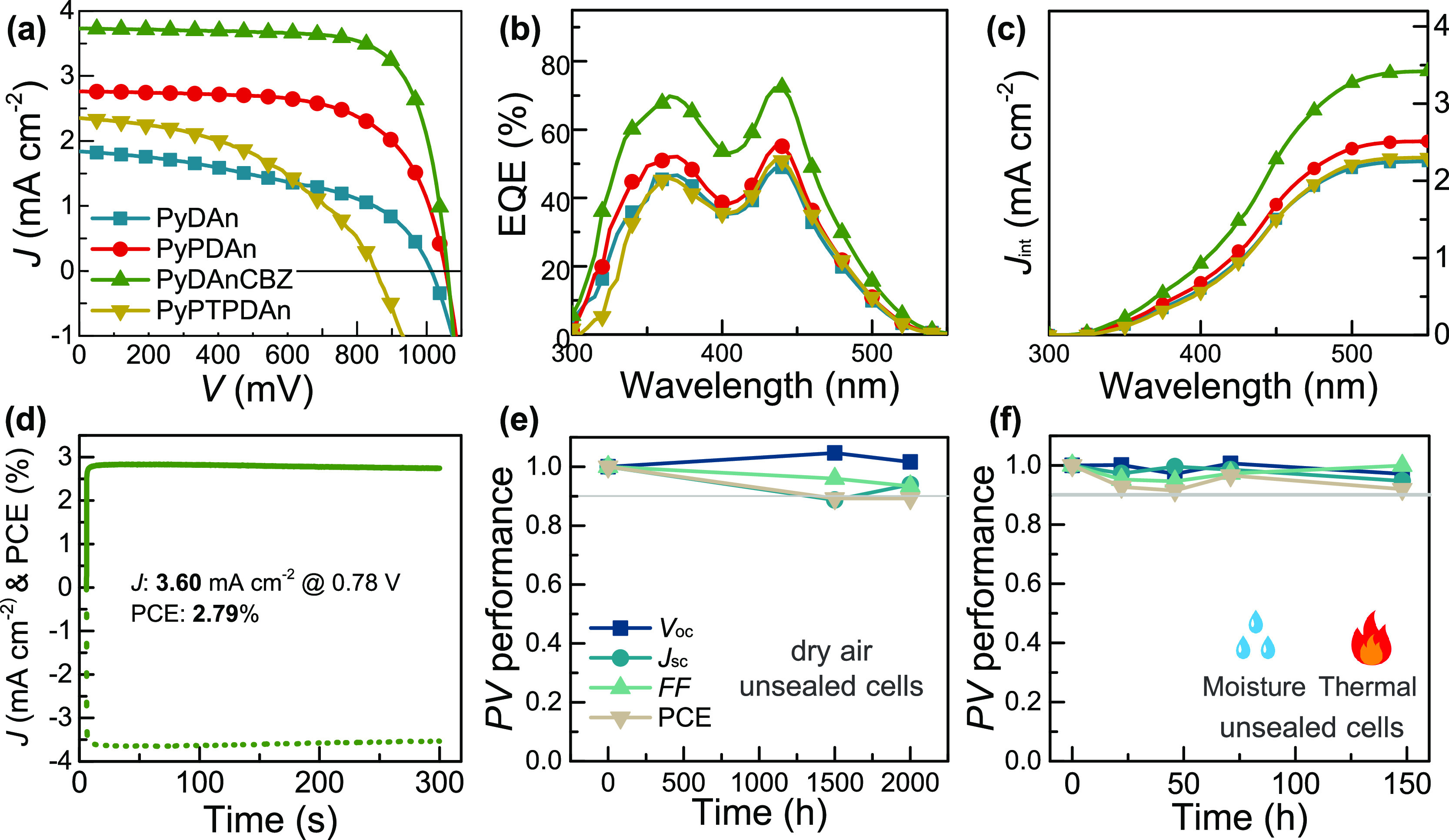
(a) *J*–*V* curves, (b) EQE spectra, and (c) corresponding integrated current density of the Cs_2_AgBiBr_6_ solar cells with different HSLs. (d) the stabilized power output of the champion device with **PyDAnCBZ** HSL. The PV performance of PSC with **PyDAnCBZ** aged under (e) dry air and (f) multi-stress (moisture and thermal) conditions.

We fabricated PSCs in the *n*-*i*-*p* structure using FTO/*bl*&*mp*-TiO_2_/Cs_2_AgBiBr_6_/HSL/Au to assess how the arm modulation of pyridine based-HSLs affected the PV performance. The cross-sectional SEM image of the PSC (Figure S8) reveals distinct layered topologies, with perovskite and **PyDAnCBZ** having thicknesses of 232 and 180 nm, respectively. The mesoporous TiO_2_ ESL with 216 nm was chosen to improve the thickness of Cs_2_AgBiBr_6_ for absorbing more light. The same precursor weight concentration was used to uniform the HSLs. The *J*–*V* curves of the optimized device measured under standard AM 1.5G irradiation are presented in Figure 3a and summarized in Table 2.

**Table 2:** PV Parameters of the PSCs with HSLs, and Diode Characteristics of the Corresponding Cells Extracted from the *J*–*V* Curves Measured under Dark Conditions

HSL	*V*_oc_ (mV)	*J*_sc_ (mA cm^–2^)	FF (%)	PCE (%)	*J* (mA cm^–2^)	*R*_s_ (Ω)	*R*_sh_ (kΩ)	*n*	*J*_0_ (mA cm^–2^)
**PyDAn**	1011.5	2.21	48.3	1.08	2.28	31	6.8	3.00	2.45 × 10^–07^
**PyPDAn**	1053.2	2.76	65.4	1.90	2.55	23	7.3	2.96	2.78 × 10^–07^
**PyDAnCBZ**	1059.0	3.73	74.0	2.92	3.48	30	11.4	2.31	9.18 × 10^–09^
**PyPTPDAn**	852.1	2.36	45.2	0.91	2.33	46	7.0	4.30	1.27 × 10^–04^

The PSCs based on **PyDAnCBZ** attained a record PCE of 2.92% measured under reverse scan (*V*_oc_ = 1059.0 mV, *J*_sc_ = 3.73 mA cm^–2^, and FF = 74.0%), whereas lower PCEs were achieved by the devices with **PyPDAn** under similar conditions. The **PyPDAn-**based PSCs delivered a PCE of 1.90% (*V*_oc_ = 1053.2 mV, *J*_sc_ = 2.76 mA cm^–2^, and FF = 65.4%). The **PyDAn**- and **PyPTPDAn**-based PSCs yielded ∼1% efficiency. The performance of the devices based on the new HSLs was in the following order: **PyDAnCBZ** > **PyPDAn** > **PyDAn** > **PyPTPDAn**. The PCE of PSCs employing **PyDAnCBZ** is higher than that of Spiro-OMeTAD and PTAA, which measured 1.6 and 1.31%, respectively (Figure S9 and Table S2 in the Supporting Information), pointing its effectiveness and suitability as a HSLs.

The reproducibility of the PSCs with the **PyDAnCBZ** was analyzed, and the PV performance is presented (Table S3) with an average PCE of ∼2.7%. Our findings demonstrate that **PyDAnCBZ** and **PyPDAn** as HSL achieved a higher *V*_oc_ and FF than those of **PyDAn** and **PyPTPDAn**. The **PyDAnCBZ**-based device showed the highest performance owing to the higher FF and *J*_sc_, which stems from the improved electrical conductivity and hole mobility and inhibited surface charge carrier recombination. The *J*–*V* curves of PSCs measured under forward and reverse scans (Figure S10 and Table S4), and the calculated *HI* values for PSCs with **PyDAn**, **PyPDAn**, **PyDAnCBZ**, and **PyPTPDAn** is 0.23, −0.07, 0.09, and −0.18, respectively. The **PyPDAn-** and **PyDAnCBZ-**based PSCs showed a reduction in hysteresis in comparison with the others, which is consistent with the device’s PV performance, and we attribute to the improved charge-transporting abilities.

The external quantum efficiency (EQE) curves displayed that the PSCs with **PyDAnCBZ** have a higher EQE value between 300–500 nm than that of the others, with higher values of 69.8 and 72.4% at 365 and 440 nm were observed respectively (Figure 3b). Additionally, the **PyDAnCBZ**-based device displayed a higher EQE value across a wider wavelength range (700–1100 nm) as shown in Figure S11. It produced an enhanced integrated current density (*J*_int_) of 3.48 mA cm^–2^ (Figure 3c), which is higher than the previously reported Cs_2_AgBiBr_6_-based devices.^40,41^ Furthermore, only Cs_2_AgBiBr_6_ is used as an absorber layer, no dye or other coabsorber is used. The outcomes are in line with those of the **PyDAnCBZ** with the highest *J*_sc_ (3.73 mA cm^–2^). The maximum power point tracking for champion **PyDAnCBZ** HSL under 1 Sun condition and electrical bias was conducted. The stabilized current density and PCE values (Figure 3d) of the device were 3.60 mA cm^–2^ and 2.79%, respectively, which is in agreement with the PV performance.

We conducted the device stability to access the reliability of lead-free solar cells. The PCEs of **PyDAnCBZ-**based devices still had around 90% of their initial value after aging at the dry box with 10–30% relative humidity and room temperature for a thousand hours (Figure 3e). The *V*_oc_ and *J*_sc_ maintained the initial value, while the FF decreased to 90% of the initial value and was the cause of the slight deterioration of device performance. Suggesting **PyDAnCBZ** and Cs_2_AgBiBr_6_ improved stability under dry air, with very little deterioration coming from the interfacial layers within the device.

The intrinsic ionic nature of double metal perovskite is prone to ion migration and impairs PV performance.^42,43^ Therefore, we assessed how the PV performed when subjected to multistress with thermal (85 °C) and ambient moisture conditions. The PCE still had 92 percent of its original value after aging around 150 h, as shown in Figure 3f. We attribute the improved reliability owing to the interaction between the pyridine core unit and Ag defects, as well as the improvement of the diffusion barrier that prevents the Ag and Br ions from migration. Importantly, the **PyDAnCBZ** possessed amorphous forms and could maintain their transporting properties under thermal aging. The phenomena constrained by **PyDAnCBZ** can be further quantified.

To elucidate the charge transport kinetics properties of the lead-free PSCs, complementary electrical characterizations were performed. Figure S12 represents the *J*–*V* curve of the PSCs measured under dark conditions, and the diode parameters extracted are represented in Table 2. The series resistance (*R*_s_) is high for all PSCs, since typical lead-based PSCs present values of *R*_s_ an order of magnitude of 1 Ω. Nevertheless, this value may be due to the resistive TiO_2_, having the HSL a low impact. The shunt resistance (*R*_sh_) value is good in all the PSCs, indicating that there are no shunt sources between the electrodes, such as pinholes or conductive phases. The dark saturation current (*J*_0_) can be associated with the carrier recombination in the cells, which correlates with the open circuit voltage.^7^ Compared to the other PSCs, the **PyDAnCBZ-**based cell shows a lower dark saturation current, correlating with the *V*_oc_ values measured in Figure 3a. Suggesting **PyDAnCBZ** employment largely mitigates the recombination, most likely in the back surface, while the other pyridine HSLs present larger surface recombination. The diode ideality factor close to 2 is a typical value for thin-film PV, which indicates that the carrier recombination occurs in the depletion area. Larger values of the ideality factor may indicate that other sources of recombination can affect the PV parameters, degrading the FF. **PyDAnCBZ-**based devices show a lower ideality factor, indicating the HSL ability to control the recombination process.

To deduce a comprehensive understanding of the enhanced PV performance, the interaction between Cs_2_AgBiBr_6_ and **PyDAnCBZ** was investigated through a series of experiments. First, we conducted X-ray diffraction (XRD) characterization to analyze the crystal structures of perovskite films with and without **PyDAnCBZ** (Figure S13). Both the films exhibited high-purity Cs_2_AgBiBr_6_ and identical crystallinity, indicating that the spin-coating of **PyDAnCBZ** on top of the perovskite films had no impact on the perovskite crystal structure.

**Figure 4:**
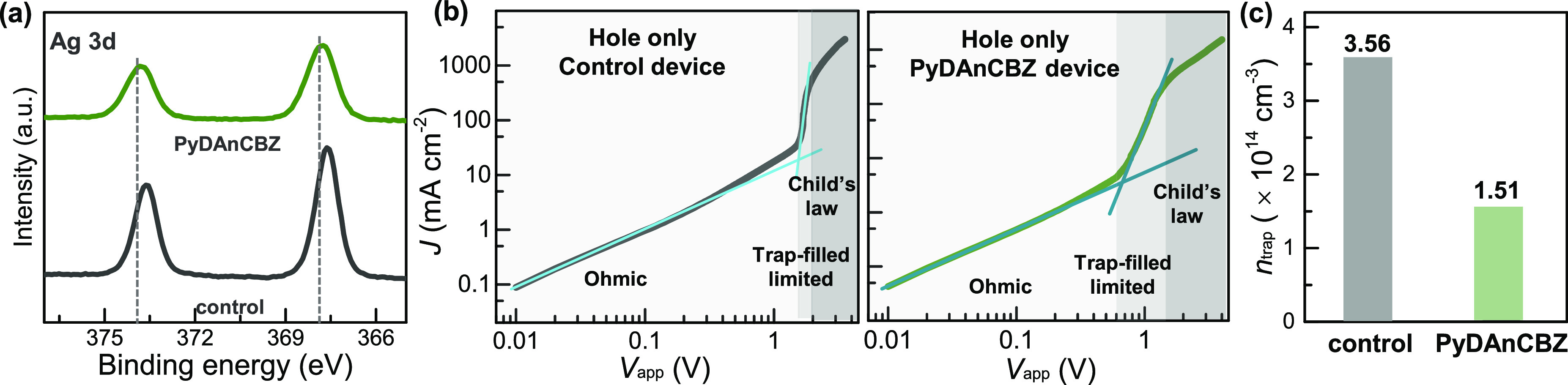
(a) XPS spectra of Ag elements of perovskite films with and without **PyDAnCBZ** HSLs. (b) SCLC curves of control and **PyDAnCBZ**-treated perovskite based on hole-only devices and (c) trap density statistic.

Further analysis was conducted using core-level X-ray photoelectron spectroscopy (XPS) to investigate the chemical states of Ag, Bi, Br, and Cs in both the control perovskite film and **PyDAnCBZ**-treated film (Figure 4a and Figure S14). The **PyDAnCBZ**-treated film showed higher binding energies for Ag 3d, attributed to the strong binding between surface silver ions and **PyDAnCBZ**. However, the characteristic peaks of Bi 4f and Br 3d showed a negligible shift after the **PyDAnCBZ** deposition. The finding suggests that the strong binding between Ag^+^ and **PyDAnCBZ** can effectively passivate surface Ag, Ag_i_, and Ag_Bi_-defects that may have arisen from ion migration during the formation of the perovskite grains.^42,44,45^

To quantitatively verify the passivation effect of **PyDAnCBZ** on defects, we utilized the space-charge-limited-current (SCLC) method to measure the hole-only device for perovskite films with and without **PyDAnCBZ**. The corresponding current–voltage curves are depicted in Figure 4b. The trap density (*N*_t_) was derived from the equation *V*_TFL_ = e*N*_t_*L*^2^/2εε_0_, where *V*_TFL_ is the trap-filling voltage, *L* represents the perovskite film thickness, and ε_0_ and ε are the vacuum permittivity and relative dielectric constant of Cs_2_AgBiBr_6_, respectively. The trap densities are calculated and summarized in Figure 4c. After the introduction of **PyDAnCBZ**, the trap densities decreased from 3.56 × 10^14^ to 1.51 × 10^14^ cm^–3^. This reduction in trap densities further suggests that the incorporation of **PyDAnCBZ** can efficiently passivate defects at the surface and grain boundaries, as well as inhibit nonradiative recombination.

**Figure 5:**
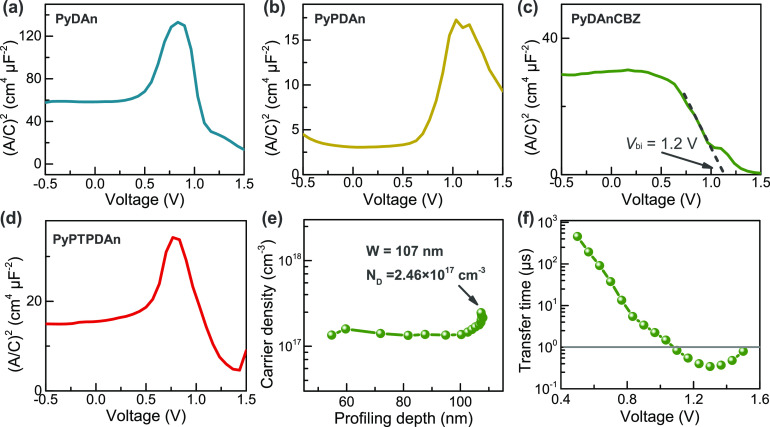
Capacitance–voltage spectra of PSCs using (a) **PyDAn**, (b) **PyPDAn**, (c) **PyDAnCBZ,** and (d) **PyPTPDAn** as HSL, with the estimation of the built-in potential when possible, (e) carrier density is calculated from the capacitance–voltage, (e) impedance spectra in the vicinity of the maximum power point voltage, and (f) transfer time calculated for the **PyDAnCBZ**.

Additionally, we performed capacitance–voltage measurements and derived Mott-Schottky plots (Figure 5a–d). In a solar cell with a diode behavior, the region of the negative slope of a Mott-Schottky plot indicates the reduction of the depletion region with the increase of the voltage, from where the built-in potential can be calculated as in the case of the **PyDAnCBZ**. However, in the other solar cells studied, a positive slope is also found, which suggests a barrier for the charge carrier has emerged, due to their poor charge carrier ability. This barrier may explain the increase of recombination found in the dark *J*–*V* curves for these cells. Since this barrier does not allow the extraction of more parameters in these cells with reliability, the rest of the measurements were made only in the **PyDAnCBZ** based solar cell. From the capacitance*–*voltage measurements, the carrier density and depletion width were calculated (Figure 5e), revealing the doping density of the Cs_2_AgBiBr_6_ is 2.46 × 10^17^ cm^–3^, while the depletion at 0 V is 107 nm. This is found to be the first bottleneck for the performance of Cs_2_AgBiBr_6_ as a light-absorbing layer, since the charge collection is limited, reducing the photocurrent that can be extracted.

The measurement of the impedance in the vicinity of the turn-on voltage (Figure S15) and we calculated the transfer time from this (Figure 5f). This parameter represents the time for a charge to recombine once is in the depletion region. A value in the order of 1 μs in the region of the maximum power point voltage is high enough to consider that losses by recombination in the junction are negligible.

**Figure 6:**
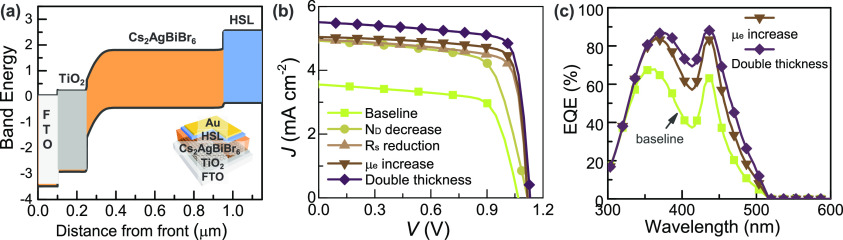
(a) Energy levels of the simulated device, (b) *J*–*V* curves of the simulated solar cell, and (c) EQE of simulated devices.

To identify the barrier pushing the Cs_2_AgBiBr_6_ solar cell performance, we simulated it using PC1D software, combining the results obtained from the characterization with the properties of Cs_2_AgBiBr_6_.^14,46^ The energy levels of the solar cell, including the FTO, TiO_2_, Cs_2_AgBiBr_6_, and HSL, are shown in Figure 6a. It can be observed that the depletion region of the solar cell simulated is in the order of 100 nm, in agreement with the value calculated by the capacitance–voltage, due to the high doping density of Cs_2_AgBiBr_6_. When simulating the device performance, it can be noted that the baseline simulation of the *J*–*V* curve and EQE calculated is close to the spectra of the **PyDAnCBZ** solar cell, as well as the PV characteristics (Table 3). Tuning of some of the key properties identified as bottlenecks was performed. First, a reduction of the doping density to a value of 10^16^ cm^–3^ allows for a higher collection of charge, increasing the current from 3.55 to 4.92 mA cm^–2^, and the PCE by 1%. A decrease in the doping density could be possible by controlling the defects and crystallinity of the Cs_2_AgBiBr_6_ films. Then, the effect of the excessive series resistance was analyzed. A reduction of an order of magnitude has a large impact on the FF, increasing from 69 to 77%.

**Table 3:** Photovoltaic Properties of the Simulated Device with the Modified Properties

parameter modified	*V*_oc_ (V)	*J*_sc_ (mA/cm^2^)	FF (%)	PCE (%)	doping density (× 10^16^ cm^–3^)	μ_e_ (cm^2^/Vs)	*R*_S_ (Ω)	thickness (μm)
baseline	1.07	3.55	71	2.7	24.6	0.08	30	0.7
*N*_D_ decrease	1.12	4.92	69	3.81	1.0	0.08	30	0.7
*R*_s_ decrease	1.12	4.96	77	4.28	1.0	0.08	3	0.7
μ_e_ increase	1.12	5.04	80	4.53	1.0	0.3	3	0.7
double thickness	1.13	5.51	79	4.90	1.0	0.3	3	1.4

Cs_2_AgBiBr_6_-based PSCs are reported to have very low electron mobility, which impacts the charge collection and avoids the possibility of increasing the thickness of the Cs_2_AgBiBr_6_ layer.^47^ Improving the electron mobility by reducing the electron traps in the perovskite layer has a small impact in thin cells, but may allow for an increase in the light-absorbing layer thickness without affecting other parameters. The effect of increasing the electron mobility and thickness was simulated, founding a significant increase in the photocurrent (Figure 6b,c). Including all the modifications mentioned, the device based on Cs_2_AgBiBr_6_ with the configuration used in this work has the potential of reaching a PCE of a 4.9%.

## 3 Experimental Section

### 3.1 Design and Synthetic of Pyridine Core-Based HSLs

#### 3.1.1 Synthesis of **PyDAn**

All the oxygen or moisture sensitive reactions were carried out under an argon atmosphere, and the reflux reactions were performed in an oil bath. A mixture of 2,6-dibromopyridine (0.5 g, 2.11 mmol), bis(4-methoxyphenyl)amine (0.965 g, 4.22 mmol), palladium acetate (0.019 g, 0.084 mmol), 1,1′-ferrocenediyl-bis(diphenylphosphine) (0.116 g, 0.211 mmol), and sodium *tert*-butoxide (0.6 g, 6.33 mmol) in toluene (20 mL) was stirred at 110 °C for 24 h. After cooling down the reaction to room temperature, the mixture was diluted with dichloromethane and washed with water. The organic layer was dried over Na_2_SO_4_ and evaporated. The crude product was purified by column chromatography (hexane/CH_2_Cl_2_ = 3/1 vol/vol) to obtain **PyDAn** (0.625 g, 70% yield) as an off-white solid.

#### 3.1.2 Synthesis of **PyPDAn**

A mixture of 4-methoxy-*N*-(4-methoxyphenyl)-*N*-(4-(4,4,5,5-tetramethyl-1,3,2-dioxaborolan-2-yl)phenyl)aniline (0.722 g, 0.84 mmol), 2,6-dibromopyridine (0.200 g, 0.84 mmol), and Pd(PPh_3_)_4_ (0.048 g, 0.042 mmol) in toluene (24 mL), ethanol (12 mL), and 2 M K_2_CO_3_ aqueous solution (3 mL) was stirred at 100 °C for 24 h under an argon atmosphere. After cooling down the reaction to room temperature, the mixture was diluted with dichloromethane and washed with water. The organic layer was dried over Na_2_SO_4_ and evaporated. The crude product was purified by column chromatography (SiO_2_, Hexane/CH_2_Cl_2_ = 1/4 vol/vol) to obtain **PyPDAn** (0.350 g, 60% yield) as an off-white solid.

#### 3.1.3 Synthesis of **PyPTPDAn**

2,6-Bromopyridine (100 mg, 0.42 mmol), PTPDAn (0.86 g, 1.05 mmol), sodium *tert*-butoxide (366.9 mg, 3.822 mmol), and dry toluene (40 mL) were mixed in a flask and purged with N_2_ for 10 min, and P(^*t*^Bu)_3_HBF_4_ (9.23 mg, 0.05 mmol) and Pd(OAc)_2_ (5.72 mg, 0.04 mmol) were added. The reaction was refluxed under N_2_ for 48 h. After completion of the reaction, monitored with TLC, the reaction was allowed to cool to room temperature, and the solution was filtered to remove insoluble solids. The filtrate was concentrated *in vacuo* and purified by column chromatography (silica gel, hexane: ethyl acetate 8:4 (v/v) as eluent) to give **PyPTPDAn** as a greenish-yellow solid with a yield of 52%. All of the characterization for the novel HSL including ^1^H/^13^C NMR spectroscopy and mass spectra are shown in the Supporting Information. The synthesis route of **PyDAnCBZ** has been reported by the reported literature from our group.^34^

### 3.2 Device Fabrication

The configuration of the PSCs is FTO/*b*&*mp*-TiO_2_/Cs_2_AgBiBr_6_/HSL/Au. The FTO glass substrates (NSG10) were first cleaned with 2% Hellmanex solution, acetone, and isopropanol. The precleaned substrates treated with UV-ozone were heated to 500 °C for over 30 min. The blocking TiO_2_ was sprayed with the solution [1 mL of titanium(IV) diisopropoxide bis(acetylacetonate) was dissolved in 19 mL of ethanol]. These substrates were kept heating for 30 min after spray deposition. The mesoporous TiO_2_ film was spin-coated at 4000 rpm for 30 s using the 0.13 g/mL 30NRD ethanol solution. The as-prepared mp-TiO_2_ was postheated at 125 °C for 15 min and 500 °C for 30 min. Then, the mp-TiO_2_ layer was impregnated in an aqueous TiCl_4_ solution (67.5 μL of TiCl_4_ in 10 mL of deionized water) at 70 °C for 1 h, followed by sintered at 500 °C for 30 min again.

The Cs_2_AgBiBr_6_ precursor solution was prepared by dissolving 2 mmol CsBr, 1 mmol AgBr, and 1 mmol BiBr_3_ in dimethyl sulfoxide (DMSO), which was stirred at 100 °C until these salts were fully dissolved in DMSO. Both the FTO/*b&mp*-TiO_2_ substrate and precursor solution were placed on the hot plate at 80 °C, and the solution was then spin-coated on the aforementioned substrate at 3000 rpm for 30 s, which was subsequently annealed at 280 °C for 5 min in the glovebox with argon. The ∼40 mg/mL **PyDAn**, **PyPDAn**, **PyDAnCBZ**, and **PyPTPDAn** with 75, 60, 30, and 25 mM were dissolved in chlorobenzene, respectively, the dopants were then added, and their molar ratios of LiTFSI and *t*-BP were fixed to 0.5 and 3.3 to the materials (Li salt solution is 520 mg mL^–1^ in acetonitrile). The precursor HSL solutions were then spin-coated atop the perovskite film at 3000 rpm for 30 s. Finally, a 70 nm thick gold electrode was thermally deposited.

### 3.3 Characterization

^1^H NMR spectra were recorded using a 400 MHz spectrometer. Chemical shifts are reported in delta expressed in parts per million (ppm) downfield from tetramethylsilane (TMS) using residual protonated solvent as an internal standard {CDCl3, 7.26 ppm}. ^13^C NMR spectra were recorded using a 100 MHz spectrometer. Chemical shifts are reported in delta (d) units, expressed in parts per million (ppm) downfield from tetramethylsilane (TMS) using the solvent as an internal standard {CDCl_3_, 77.16 ppm}. The ^1^H NMR splitting patterns have been described as “s, singlet; d, doublet; t, triplet; and m, multiplet”. HRMS were recorded on a Bruker-Daltonics micrOTOF-Q II mass spectrometer. UV–visible absorption spectra of all compounds were recorded on a PerkinElmer Lamba 35 UV–visible spectrophotometer in dichloromethane solution. Cyclic voltammograms were recorded on a CHI620D electrochemical analyzer (potentiostat) in dichloromethane solvent using glassy carbon as the working electrode, Pt wire as the counter electrode, and saturated calomel electrode (SCE) as the reference electrode. The scan rate was 100 mV s^–1^ for cyclic voltammetry. A solution of tetrabutylammonium hexafluorophosphate (TBAPF_6_) in dichloromethane (0.1 M) was used as the supporting electrolyte.

Thermogravimetric analysis (TGA) was carried out using Mettler Toledo TGA/DSC 1 at a heating rate of 5 °C min^–1^ under a nitrogen atmosphere. The glass transition temperature for the pyridine-based HSLs was recorded by different scanning calorimetry (Pekin Elmer Diamond DSC) with a scan rate of 10 min^–1^ under N_2_ gas flow. The second curve was analyzed. The SEM images were recorded by Hitachi S-4800.

EPR spectra were performed at room temperature using a Bruker ELEXSYS E500 spectrometer operating at the X-band. The spectrometer was equipped with a superhigh-Q resonator ER-4123-SHQ. Toluene solutions of undoped and doped samples were placed in quartz tubes, and spectra were recorded using typical modulation amplitudes of 1.0 G at a frequency of 100 kHz. The magnetic field was calibrated by an NMR probe, and the frequency inside the cavity (∼9.4 GHz) was determined with an integrated MW-frequency counter. The radical concentration was evaluated by integrating the EPR spectrum twice and by comparing it with a standard CuSO_4_·5H_2_O sample. Data were collected and processed using the Bruker Xepr suite.

The *J*–*V* curves of the solar cells with an active area of 0.09 cm^2^ controlled by a metal mask were measured under AM 1.5G illumination that was provided by a 3A grade solar simulator (Newport). The forward scan range is 0–1.3 V, and the reverse scan range is from 1.3 to 0 V (scan rate: 10 mV s^–1^). The EQE was measured by a 150 W Xenon lamp (Newport) attached to IQE200B (Oriel) motorized 1/4m monochromator as the light source. The impedance of devices was tested by Bio-logic SP-300 under different voltages in different frequencies. The devices with structures FTO/HSL/Au and FTO/PEDOT:PSS/HSL/Au were fabricated and measured under dark conditions for calculating conductivity and mobility.

Impedance and capacitance–voltage measurements were performed inside a Faraday box with a Biologic impedance analyzer, following the reported literature,^48,49^ by applying a 20 mV perturbation in a frequency range from 1 GHz to 100 Hz, and applied constant voltage from −0.5 to 1.5 V. EC-lab software was used to fit the equivalent circuits.

## 4 Conclusions

We developed and assessed four pyridine-based small molecules as a *p*-type selective material, investigated how the arm size affected the electro-optical properties, and deduced that arm modulation had an impact. Lead-free solar cells made with **PyDAnCBZ** outperformed the others. In comparison to the other compounds, **PyDAnCBZ** has a hyperfine coupling constant, and peak-to-peak linewidth is substantially larger, which suggests a higher spin density close to the pyridine core. Electrical measurements suggest that **PyDAnCBZ** facilitates improved charge carrier transport. The solar cells with **PyDAnCBZ** as a hole selective layer also displayed excellent thermal and moisture stability. Further, we suggested the roadmap through the photovoltaic performance simulation that, with fine-tuning, Cs_2_AgBiBr_6_ solar cells can increase their power conversion efficiency to 4.9%.

## Supporting Information Available

Materials synthesis its spectroscopic and structural characterization, and the device electrical properties (PDF)
