# Supplementary material for: Molecular Tailoring of Pyridine Core-Based Hole Selective Layer for Lead Free Double Perovskite Solar Cells Fabrication
Source: ACS Appl Energy Mater. 2023 Jul 17;6(15):7955–64. doi: 10.1021/acsaem.3c01027 (PMC10428164; doi:10.1021/acsaem.3c01027)
Supplement: Supplementary file 1 — ae3c01027_si_001.pdf [file ae3c01027_si_001.pdf]

Supporting information for

Molecular Tailoring of Pyridine Core-Based Hole Selective Layer for Lead Free Double Perovskite Solar Cells Fabrication

Peng Huang[a,f], Manju Sheokand[b], David Payno[a], Samrana Kazim[a,e], Luis Lezama[c], Mohammad Khaja Nazeeruddin[d], Rajneesh Misra[b], and Shahzada Ahmad[a,e,*]*

[a]BCMaterials, Basque Center for Materials, Applications and Nanostructures, Martina Casiano, UPV/EHU Science Park, 48940, Leioa, Spain

Email: shahzada.ahmad@bcmaterials.net

[b]Department of Chemistry, Indian Institute of Technology, 453552, Indore, India

Email: rajneeshmisra@iiti.ac.in

[d]Departamento de Química Inorgánica, Facultad de Ciencia y Tecnología, Universidad del País Vasco, UPV/EHU, Sarriena s/n, 48940, Leioa, Spain

[d]Group for Molecular Engineering of Functional Materials, Institute of Chemical Sciences and Engineering, École Polytechnique Fedérale de Lausanne, 1951 Sion, Switzerland

[e] IKERBASQUE, Basque Foundation for Science, 48009, Bilbao, Spain

[f]Research Institute of Frontier Science, Southwest Jiaotong University, 610031, Chengdu, China

Experimental section.

Synthesis of intermediate 1c:

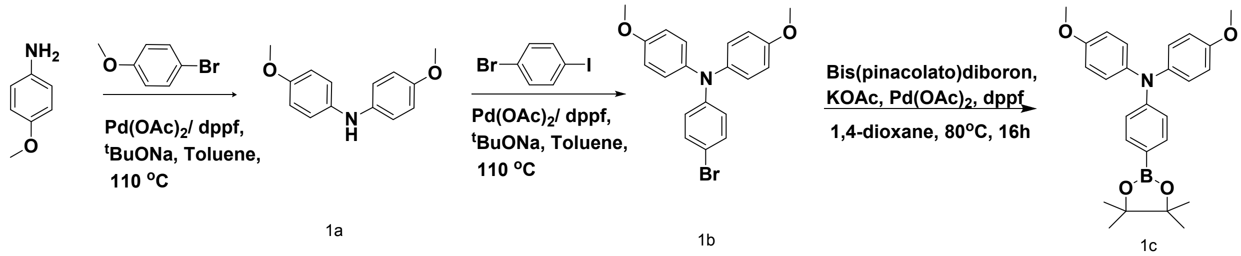

Scheme S1. Synthetic route to intermediate **1c.**

Bis(4-methoxyphenyl)amine (1a). A mixture of 4-bromoanisole (1.496 g, 8.00 mmol), *p*-anisidine (0.985 g, 8.00 mmol), palladium acetate (0.036 g, 0.16 mmol), 1,1′-ferrocenediyl-bis(diphenylphosphine) (0.177 g, 0.32 mmol) and sodium *tert*-butoxide (1.153 g, 12.00 mmol) in toluene (40 mL) was stirred at 110 °C for 24 h. After cooling down the reaction to room temperature, the mixture was diluted with dichloromethane and washed with water. The organic layer was dried over Na_2SO_4 and evaporated. The crude product was purified by column chromatography (Hexane/CH_2Cl_2 = 1/1 vol/vol) to obtain **1a** (1.291 g, 70.3% yield) as an off-white solid. ^{1}H-NMR ($CDCl_3$): δ 7.01-6.97 (m, 4H); 6.86-6.82 (m, 4H); 6.77 (s, br, 1H); 3.76 (s, 6H) ppm.

4-Bromo-*N*,*N*-bis(4-methoxyphenyl)aniline (1b). A mixture of **1a** (1.355 g, 5.91 mmol), 1-Bromo-4-iodobenzene (1.671 g, 5.91 mmol), palladium acetate (0.066 g, 0.29 mmol), 1,1′-ferrocenediyl-bis(diphenylphosphine) (0.323 g, 0.58 mmol) and sodium *tert*-butoxide (0.852 g, 8.87 mmol) in toluene (30 mL) was stirred at 110 °C for 24 h. After cooling down the reaction to room temperature, the mixture was diluted with dichloromethane and washed with water. The organic layer was dried over Na_2SO_4 and evaporated. The crude product was purified by column chromatography (SiO_2, Hexane/CH_2Cl_2 = 9/1 vol/vol) to obtain **1b** (1.723 g, 75.9% yield) as a white solid. ^{1}H-NMR ($CDCl_3$): δ 7.26 (d, J = 8.8 Hz, 2H); 7.05 (d, J = 8.9 Hz, 4H); 6.85 (d, J = 8.9 Hz, 4H); 6.81 (d, J = 8.8 Hz, 2H); 3.82 (s, 6H) ppm. ^{13}C-NMR ($CDCl_3$): δ 159.6, 135.6, 133.8, 131.0, 129.7, 124.9, 119.4, 116.9, 116.6, 116.2, 55.6 ppm.

4-Methoxy-*N*-(4-methoxyphenyl)-*N*-(4-(4,4,5,5-tetramethyl-1,3,2-dioxaborolan-2-yl)phenyl)aniline (1c). A mixture of **1b** (1.100 g, 2.86 mmol), bis(pinacolato)diboron (1.089 g,

4.29 mmol), palladium acetate (0.033 g, 0.14 mmol), 1,1′-ferrocenediyl-bis(diphenylphosphine) (0.161 g, 0.29 mmol) and potassium acetate (0.571 g, 5.72 mmol) in 1,4-dioxane (10 mL) was stirred at 60 °C for 24 h. After cooling down the reaction to room temperature, the mixture was diluted with dichloromethane and washed with water. The organic layer was dried over Na_2SO_4 and evaporated. The crude product was purified by column chromatography (SiO_2, Hexane/CH_2Cl_2 = 1/1 vol/vol) to obtain **1c** (1.003 g, 73.3% yield) as a white solid. ^{1}H-NMR ($CDCl_3$): δ 7.57-7.54 (m, 2H); 7.11-7.08 (m, 4H); 6.96-6.93 (m, 4H); 6.81-6.78 (m, 2H); 3.82 (s, 6H); 1.32 (s, 12H) ppm. ^{13}C-NMR ($CDCl_3$): δ 156.8, 151.6, 140.1, 135.7, 127.4, 117.6, 114.8, 83.1, 54.8, 24.3 ppm.

*Synthesis of **PTPDAn***. A mixture of 4-methoxy-N-(4-methoxyphenyl)-N-(4-(4,4,5,5-tetramethyl-1,3,2-dioxaborolan-2- yl)phenyl)aniline (0.485 g, 1.127 mmol), 3,7-dibromo-10H-phenothiazine (0.200 g, 0.563 mmol) and $Pd(PPh_3)_4$ (0.032 g, 0.028 mmol) in tetrahydrofuran (20 mL), and 2M K_2CO_3 aqueous solution (5 mL) was stirred at 80 °C for 24 h under an argon atmosphere. After cooling down the reaction to room temperature, the mixture was diluted with dichloromethane and washed with water. The organic layer was dried over Na_2SO_4 and evaporated. The crude product was purified by column chromatography (SiO_2, Hexane/CH_2Cl_2 = 1/1 vol/vol) to obtain PTPDAn (0.453 g, 64% yield) as a yellow solid. ^{1}H-NMR ($CDCl_3$): δ 7.39 (s, 2H); 7.31 (d, J = 8, 4H); 7.23(d, J = 8, 2H); 7.08 (d, J = 8Hz, 8H); 6.96 (d, J = 8Hz, 4H); 6.85 (d, J = 8Hz, 8H); 3.80 (s, 12 H) ppm. HRMS (ESI): calcd. for $C_{52}H_{44}N_3O_4S$ [M]+: 805.2974. Found: 805.2969.

Copies of NMR and mass of new compounds

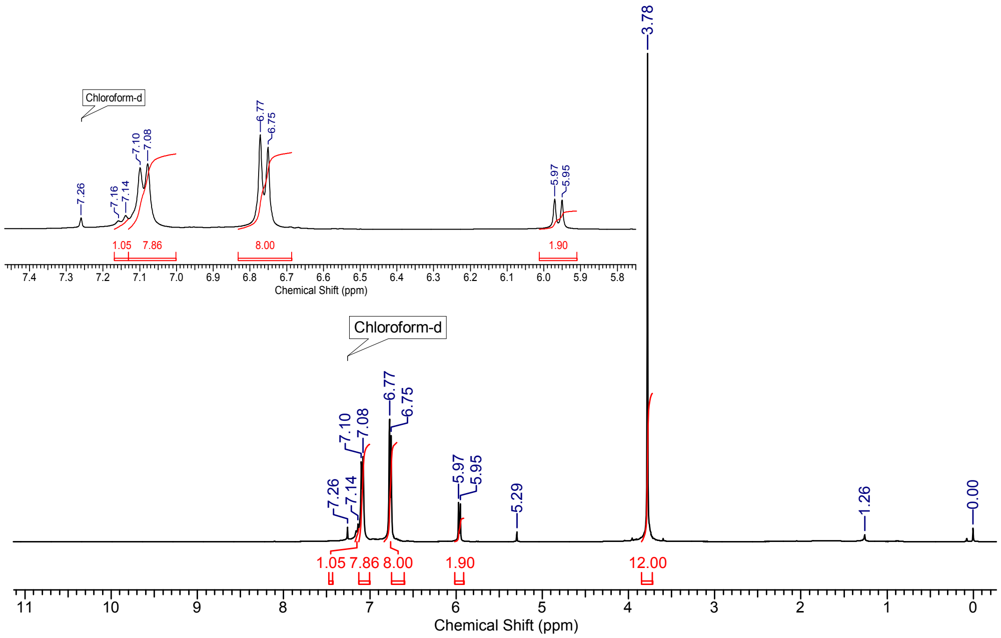

^{1}H NMR of PyDAn

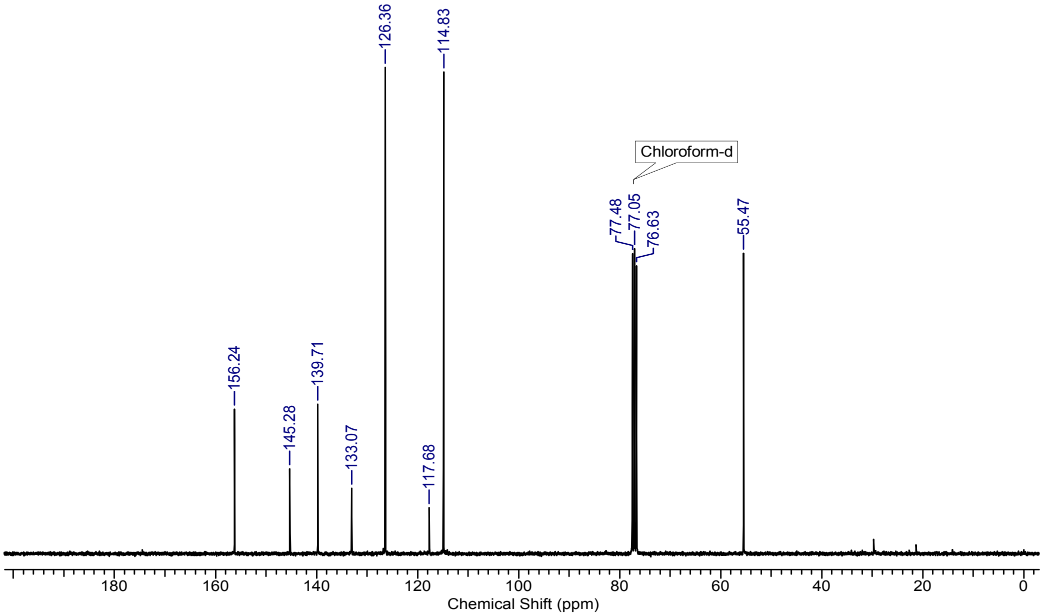

^{13}C NMR of PyDAn

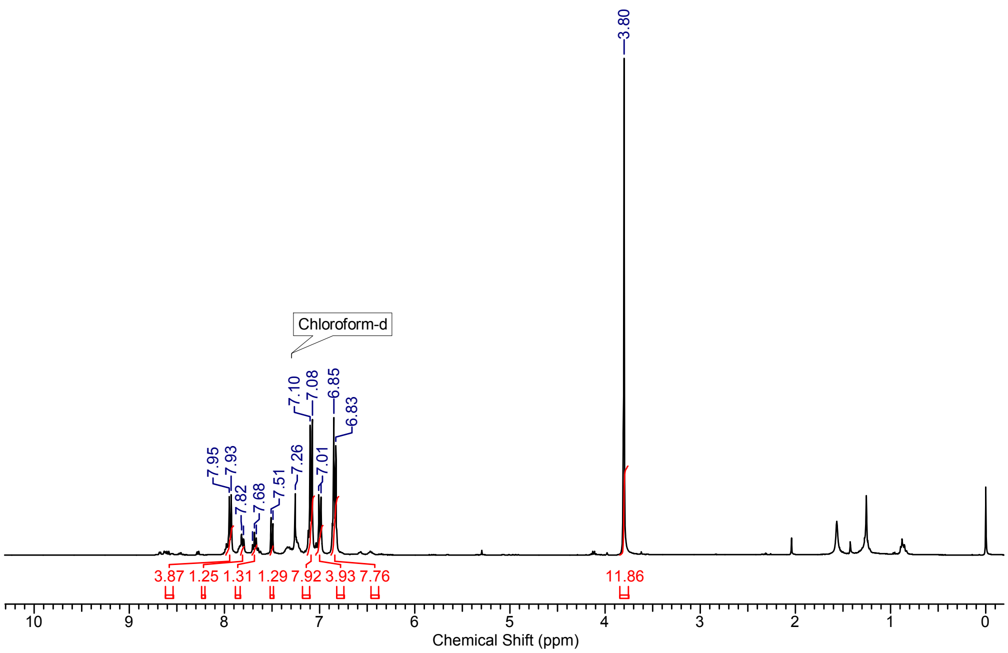

^{1}H NMR of PyPDAn

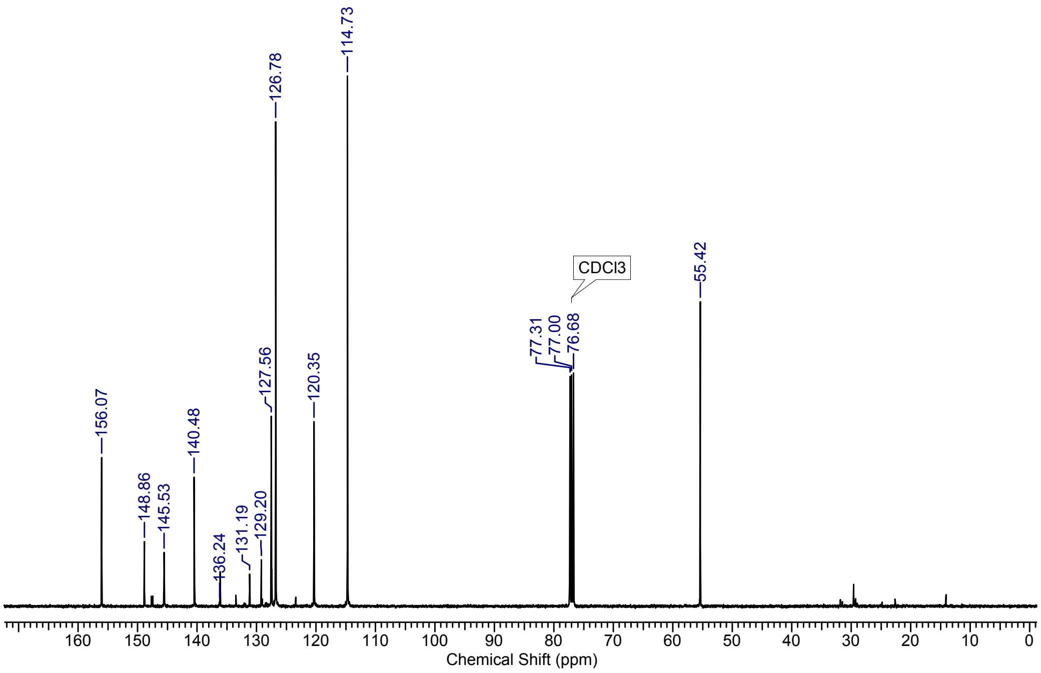

^{13}C NMR of **PyPDAn**

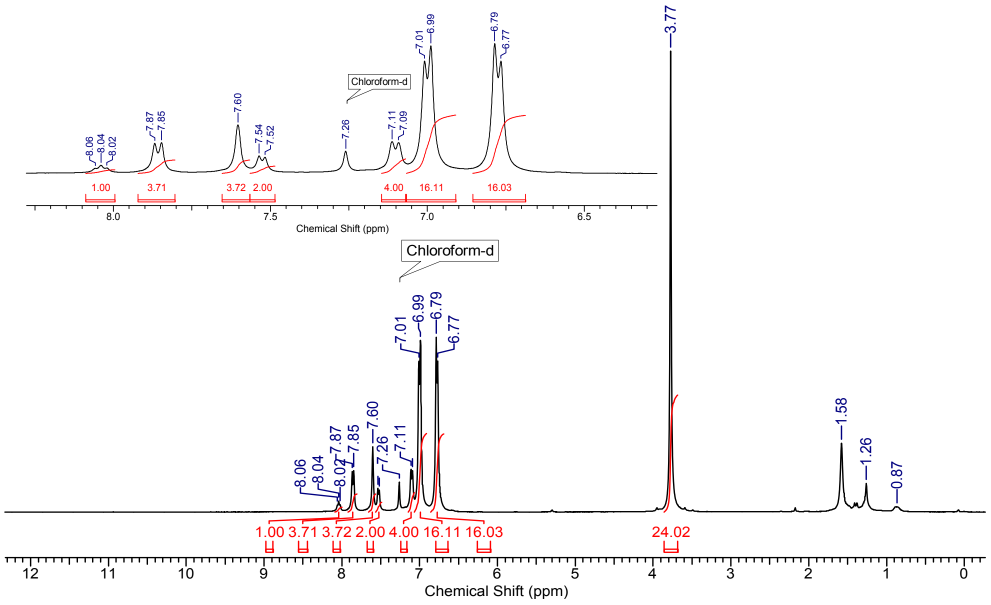

^{1}H NMR of **PyDAnCBZ**

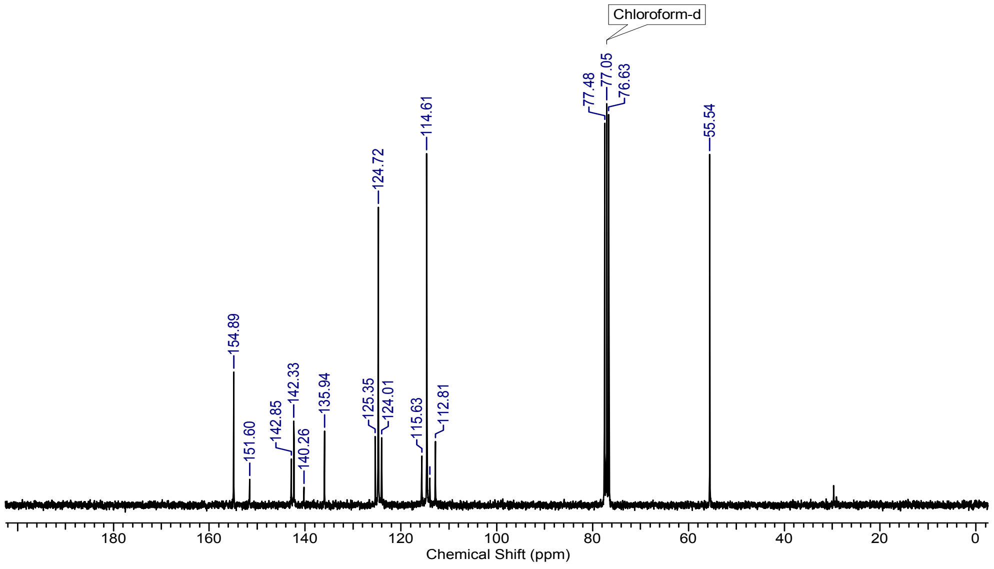

^{13}C NMR of **PyDAnCBZ**

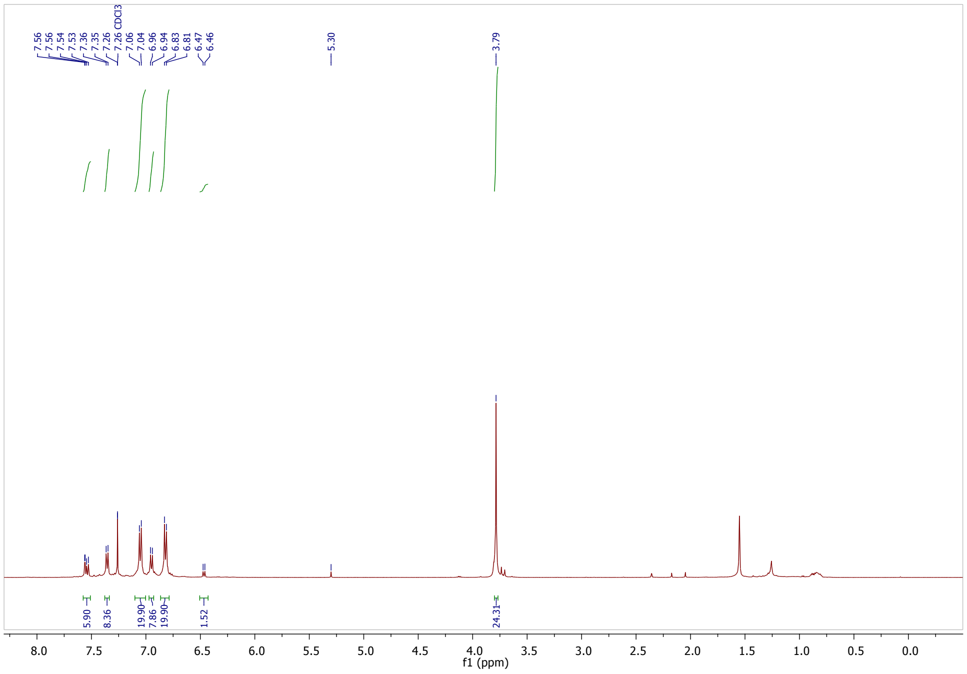

^{1}H NMR of **PyPTPDAn**

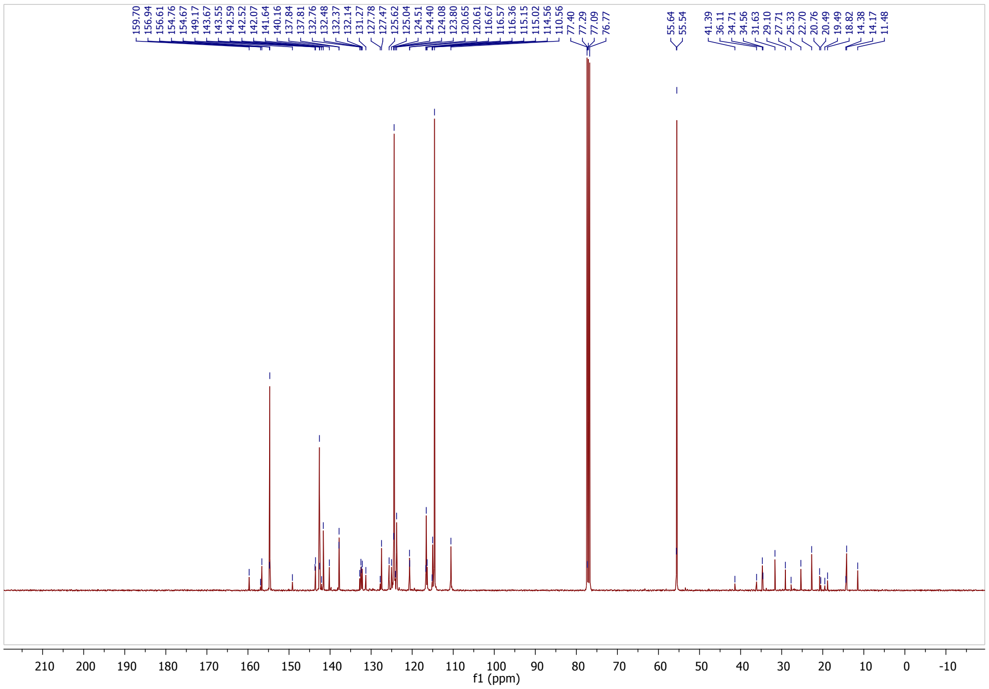

^{13}C NMR of **PyPTPDAn**

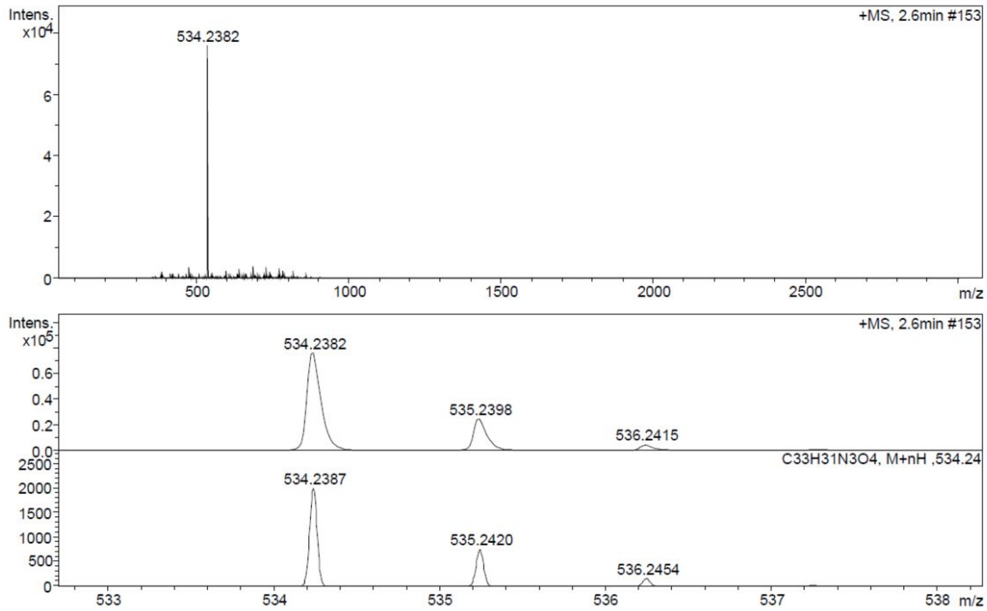

HRMS **of PyDAn**

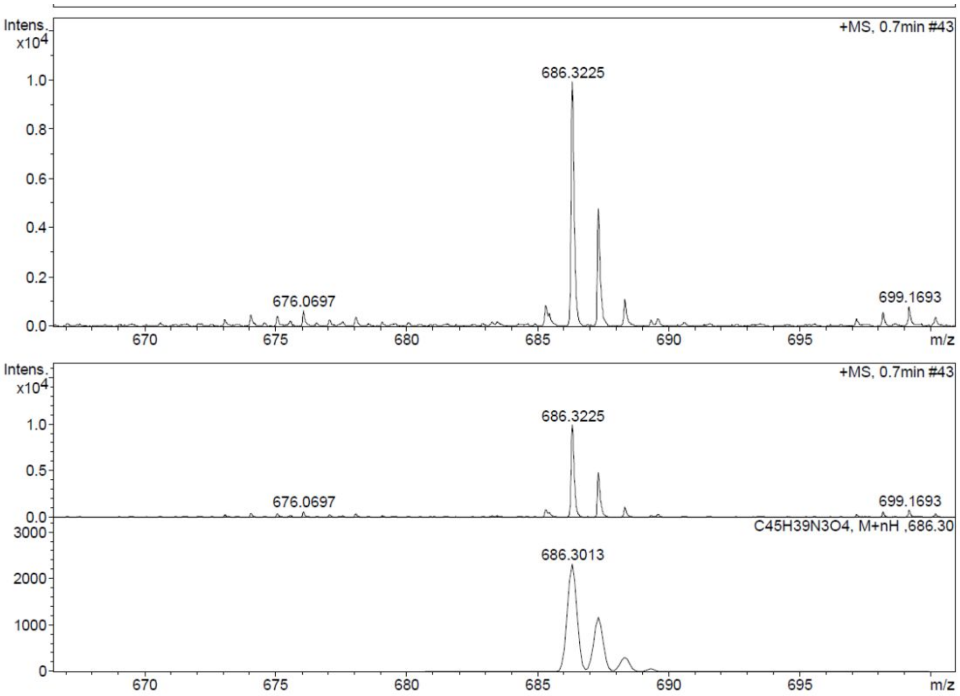

HRMS of **PyPDAn**

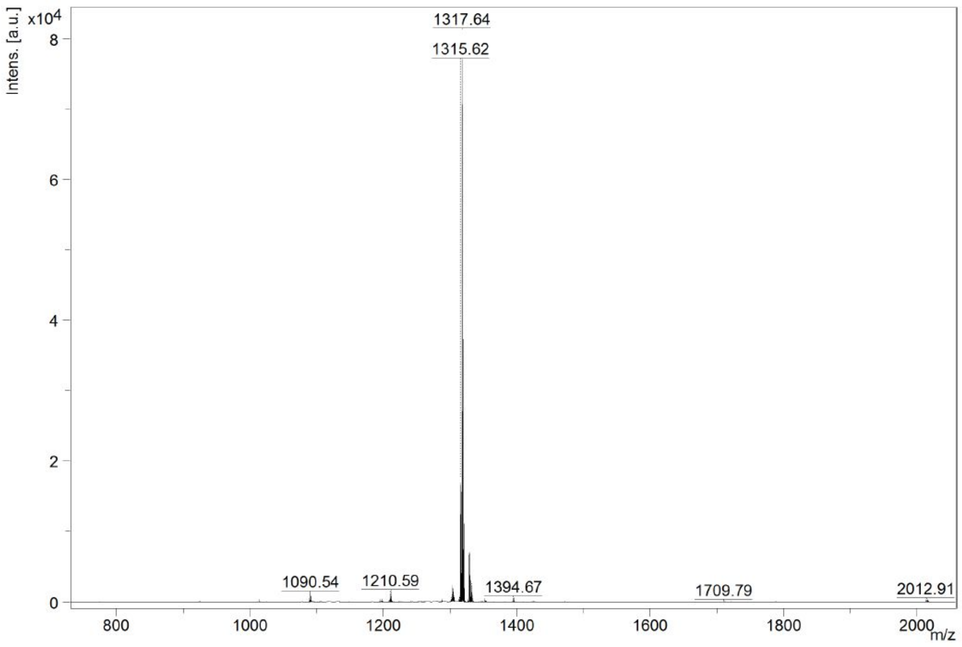

MALDI of **PyDAnCBZ**

Figure S1 ^{1}H NMR, ^{13}C NMR spectroscopy, and HRMS spectra of the hole selective materials.

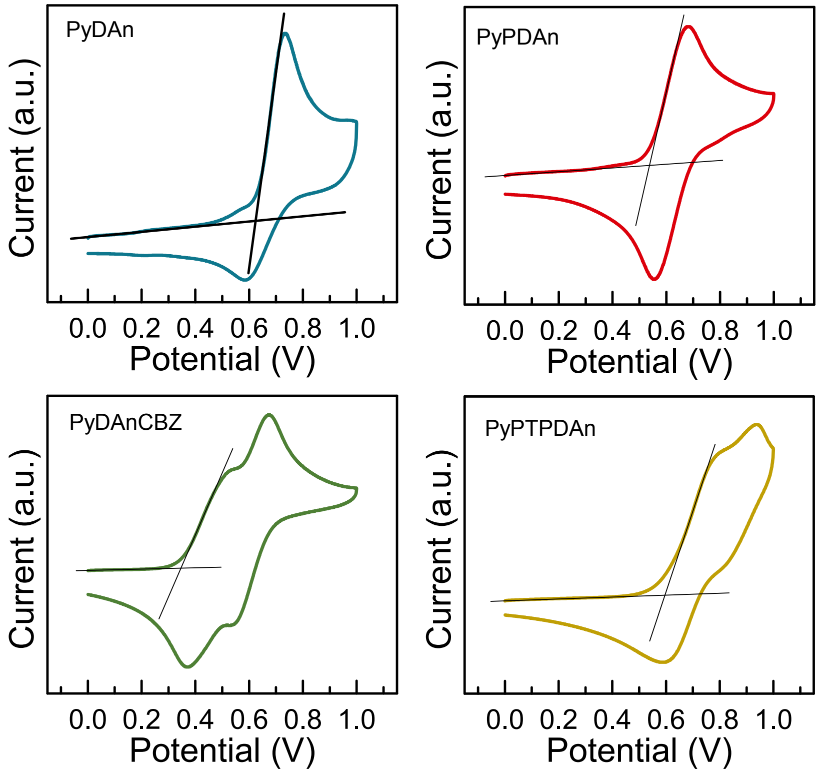

Figure S2. The cyclic voltammograms of the HSLs. The intersection of the two lines is the initial oxidation potential.

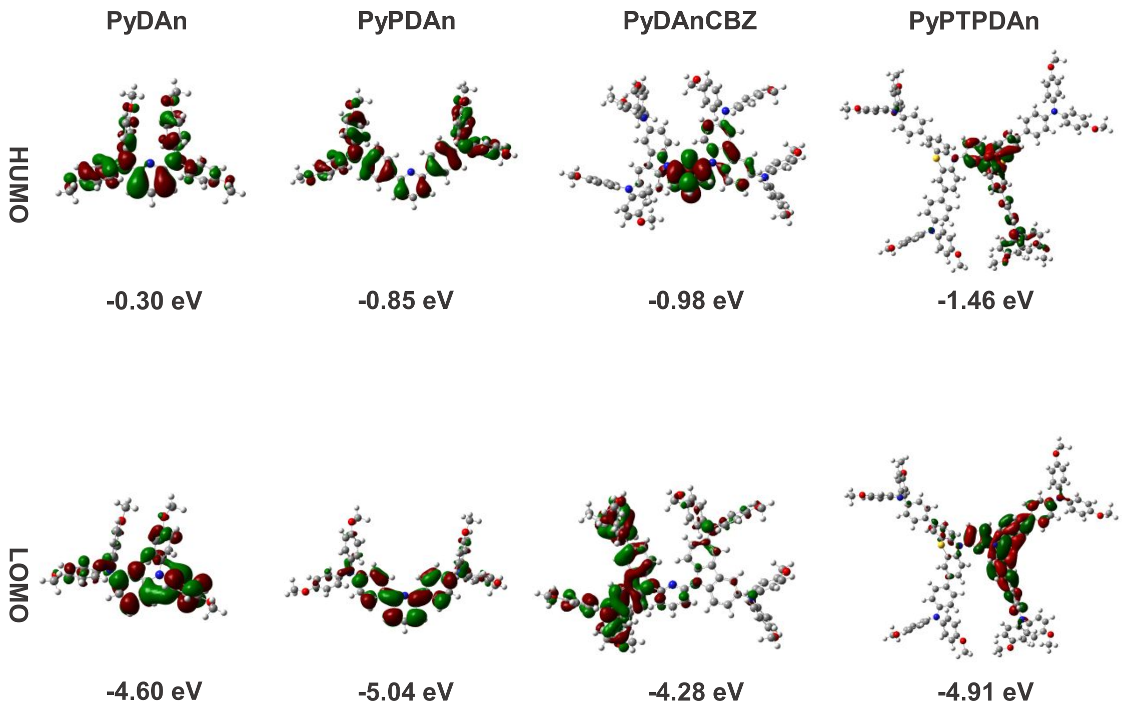

Figure S3. Energy level diagram showing the E_{HOMO} and E_{LUMO} levels of **PyDAn**, **PyPDAn**, **PyDAnCBZ**, and **PyPTPDAn**, respectively.

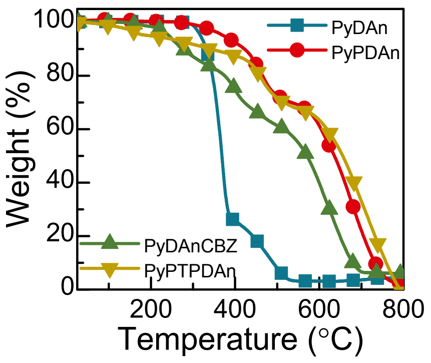

Figure S4. Thermogravimetric analysis curves.

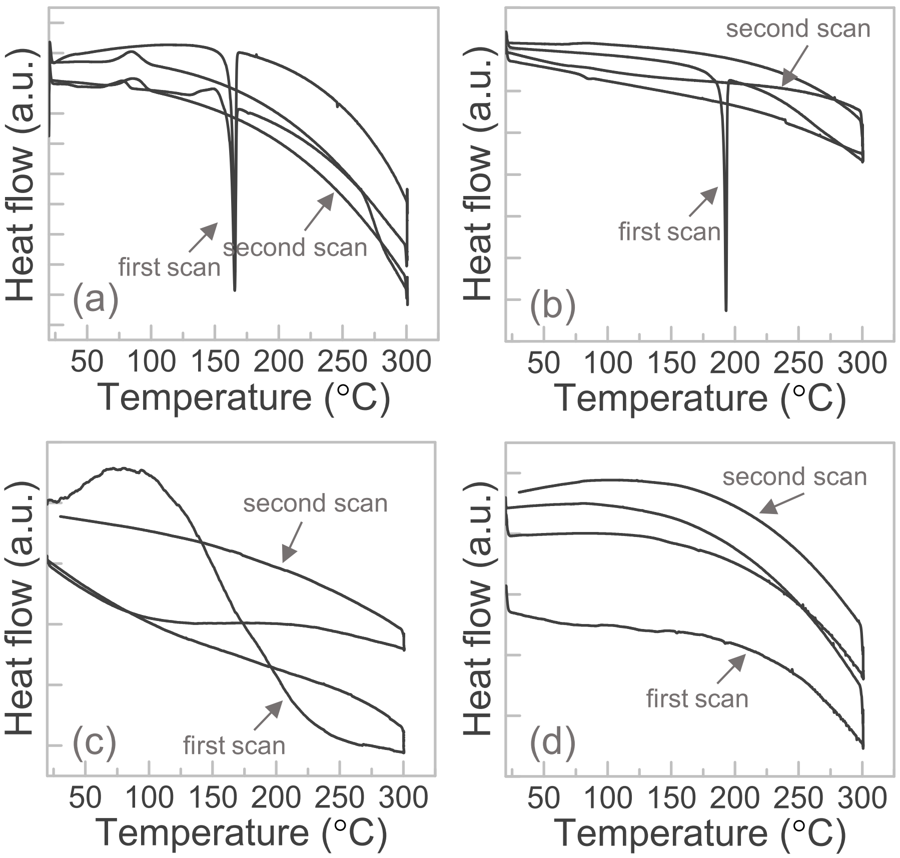

Figure S5. The DSC scan of pyridine-based HSLs with different arms (a) **PyDAn**, (b) **PyPDAn**, (c) **PyDAnCBZ**, and (d)**PyPTPDAn**.

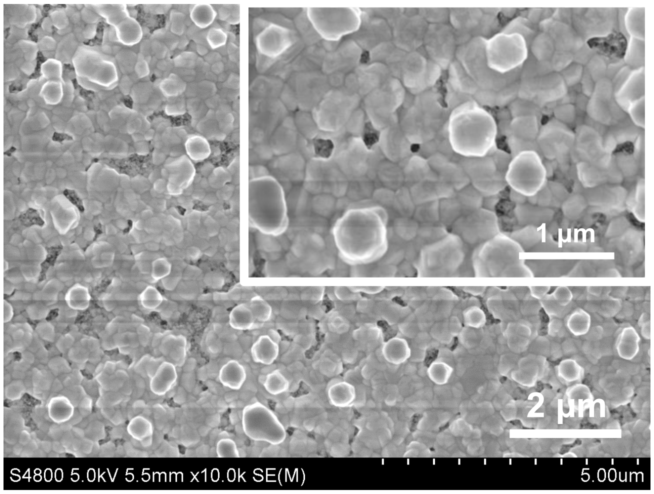

Figure S6. Top-view SEM images for the $Cs_2AgBiBr_6$ on the substrate of FTO/b&mp-TiO_2.

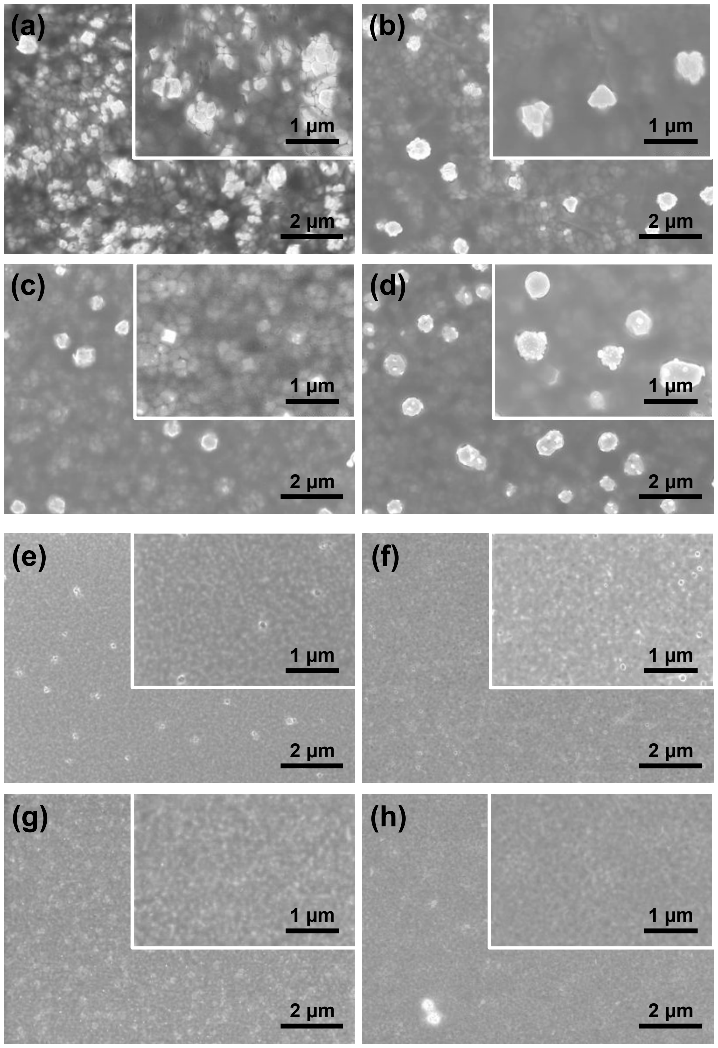

Figure S7. Top-view SEM images for $Cs_2AgBiBr_6$ films with (a) **PyDAn**, (b) **PyPDAn**, (c) **PyDAnCBZ**, and (d) **PyPTPDAn**, for the HSLs with dopants on flat substrates with (e) **PyDAn**, (f) **PyPDAn**, (g) **PyDAnCBZ**, and (h) **PyPTPDAn**.

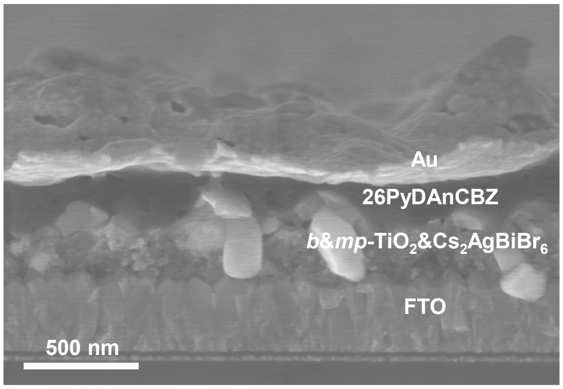

Figure S8. The cross-sectional SEM images of the $Cs_2AgBiBr_6$ solar cells with the **PyDAnCBZ** HSL.

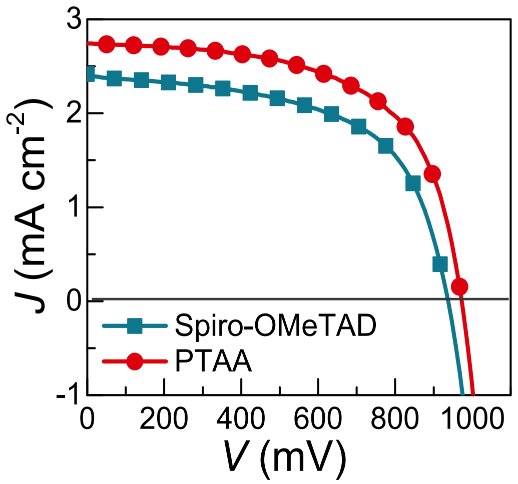

Figure S9. *J*-*V* curves of the $Cs_2AgBiBr_6$ solar cells with typical HSLs.

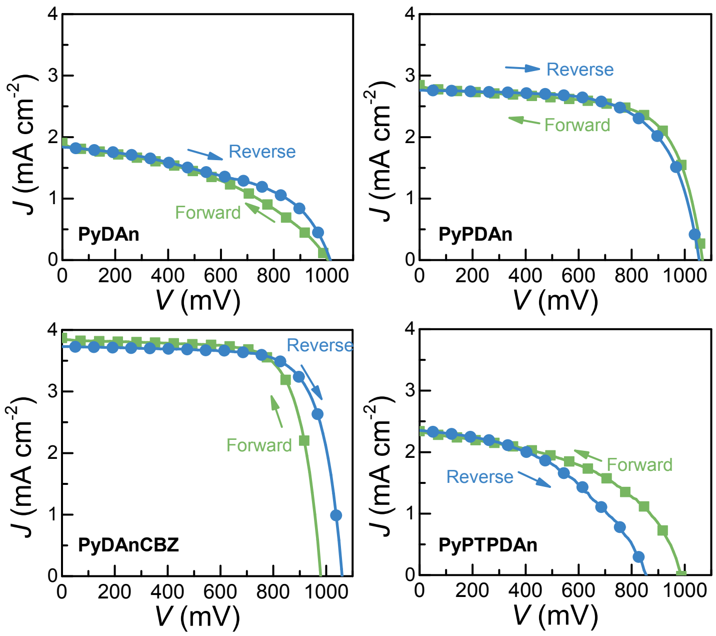

Figure S10. The hysteresis phenomena of the $Cs_2AgBiBr_6$ solar cells with different hole selective layers. The hysteresis index (*HI*), which is calculated using the equation: $HI = [J_{RS}(0.8V_{oc}) - J_{FS}(0.8V_{oc})]/J_{RS}(0.8V_{oc})$, was also used to analyze the hysteresis behavior for the aforementioned PSCs, where $J_{RS}(0.8V_{oc})$ and $J_{FS}(0.8V_{oc})$ denote the current density of *J*−*V* curves obtained by RS and FS at 80% of V_{oc}, respectively.

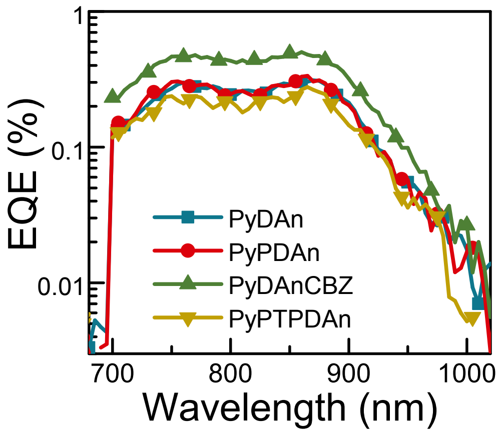

Figure S11. EQE spectra of the $Cs_2AgBiBr_6$ solar cells with **PyDAn**, **PyPDAn**, **PyDAnCBZ**, and **PyPTPDAn**, respectively.

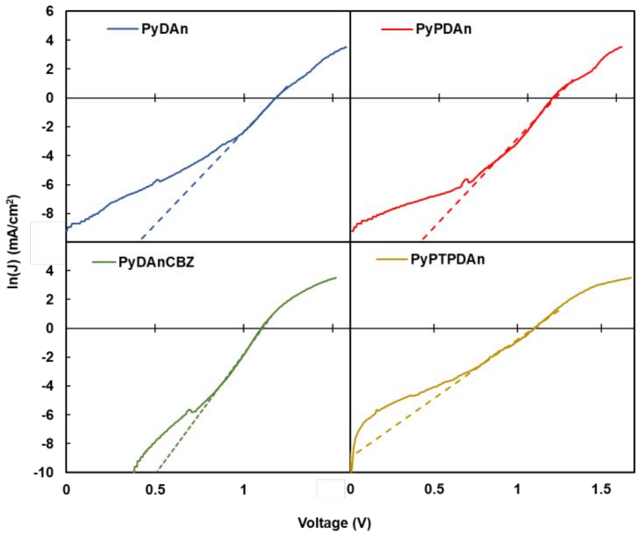

Figure S12. Dark *J-V* curves for the PSCs with different HSL, with the fit of the diode ideality factor in the range of the turn-on voltage.

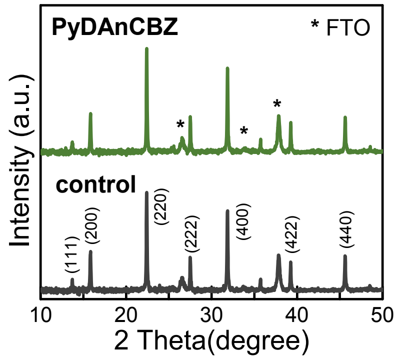

Figure S13. XRD patterns of perovskite films with and without PyDAnCBZ as HSL.

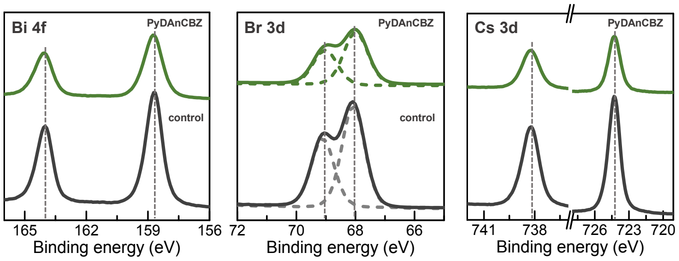

Figure S14. XPS spectra of Bi 4f, Br 3d, and Cs 3d elements of perovskite films with and without PyDAnCBZ.

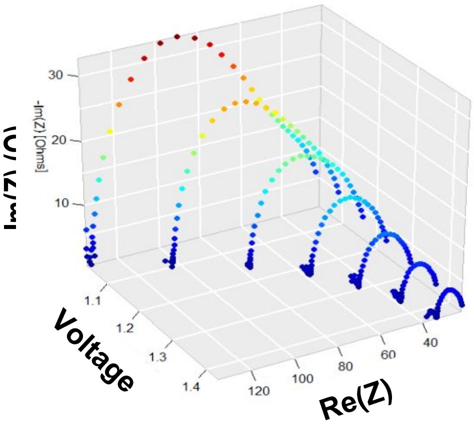

Figure S15. The Nyquist plot vs. bias voltage measured under the dark condition.

Table S1. EPR parameters for 25mM toluene solutions of doped samples

HSL	g	a^N (Gauss)	ΔB_{pp} (Gauss)	spin/mol
**PyDAn**	2.0044	5.9	3.9	7.9×10^{17}
**PyPDAn**	2.0040	6.6	4.4	2.2×10^{16}
**PyDAnCBZ**	2.0037	6.8	4.8	6.1×10^{17}
**PyPTPDAn**	2.0043	6.2	3.5	8.4×10^{17}

Table S2. The photovoltaic parameters of the $Cs_2AgBiBr_6$-based device with typical HSL.

**HSL**	V_{oc} (mV)	J_{sc} (mA/cm^2)	FF (%)	PCE (%)

**Spiro-OMeTAD**	936.6	2.40	58.4	1.31
**PTAA**	972.3	2.75	60.0	1.60

Table S3. The photovoltaic parameters of $Cs_2AgBiBr_6$ solar cells with **PyDAnCBZ** as HSL.

No.	V_{oc} (mV)	J_{sc} (mA/cm^2)	FF (%)	PCE (%)
1	1023.8	3.49	75.2	2.68
2	1059.0	3.73	74.0	2.92
3	1065.0	3.70	73.5	2.90
4	1023.8	3.74	73.2	2.80
5	1010.8	3.54	71.9	2.57
6	946.2	3.58	71.8	2.44
7	981.2	3.88	71.6	2.72
8	985.0	3.78	67.8	2.52
9	1011.9	3.91	67.5	2.67
10	1034.3	3.65	67.0	2.53
ave. values	1014.1 ± 36.1	3.70 ± 0.14	71.3 ± 2.9	2.68 ±0.17

Table S4 The $Cs_2AgBiBr_6$ solar cells with different hole-selective layers, forward and reverse scan measurements.

HSL	Scan directions	V_{oc} (mV)	J_{sc} (mA/cm^2)	FF (%)	PCE (%)
PyDAn	FS	1000.0	2.25	41.6	0.94
PyDAn	RS	1011.5	2.21	48.3	1.08
PyPDAn	FS	1066.7	2.81	66.6	2.00
PyPDAn	RS	1053.2	2.76	65.4	1.90
PyDAnCBZ	FS	1028.8	3.79	74.5	2.91
PyDAnCBZ	RS	1059.0	3.73	74.0	2.92
PyPTPDAn	FS	984.6	2.33	48.5	1.11
PyPTPDAn	RS	852.1	2.36	45.2	0.91
